# Controlled Drug Delivery Systems: Current Status and Future Directions

**DOI:** 10.3390/molecules26195905

**Published:** 2021-09-29

**Authors:** Shivakalyani Adepu, Seeram Ramakrishna

**Affiliations:** Center for Nanofibers and Nanotechnology, National University of Singapore (NUS), 21 Lower Kent Ridge Rd, Singapore 119077, Singapore

**Keywords:** controlled release dosage forms, pharmacokinetics, nano-drug delivery, smart and stimuli-responsive delivery, intelligent biomaterials

## Abstract

The drug delivery system enables the release of the active pharmaceutical ingredient to achieve a desired therapeutic response. Conventional drug delivery systems (tablets, capsules, syrups, ointments, etc.) suffer from poor bioavailability and fluctuations in plasma drug level and are unable to achieve sustained release. Without an efficient delivery mechanism, the whole therapeutic process can be rendered useless. Moreover, the drug has to be delivered at a specified controlled rate and at the target site as precisely as possible to achieve maximum efficacy and safety. Controlled drug delivery systems are developed to combat the problems associated with conventional drug delivery. There has been a tremendous evolution in controlled drug delivery systems from the past two decades ranging from macro scale and nano scale to intelligent targeted delivery. The initial part of this review provides a basic understanding of drug delivery systems with an emphasis on the pharmacokinetics of the drug. It also discusses the conventional drug delivery systems and their limitations. Further, controlled drug delivery systems are discussed in detail with the design considerations, classifications and drawings. In addition, nano-drug delivery, targeted and smart drug delivery using stimuli-responsive and intelligent biomaterials is discussed with recent key findings. The paper concludes with the challenges faced and future directions in controlled drug delivery.

## 1. Introduction

**Figure 1:**
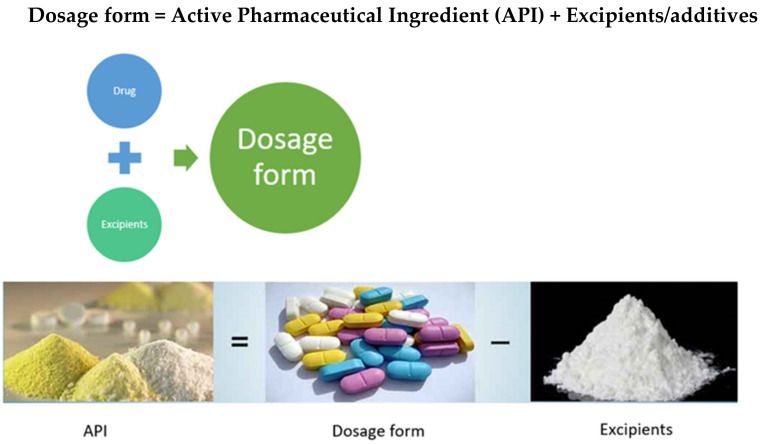
Dosage form composition.

A drug (API) is a substance (recognized in official pharmacopoeia) intended for use in the diagnosis, cure, mitigation, treatment, or prevention of disease as per the FDA. Drug delivery is a technique of delivering medication to a patient in such a manner that specifically increases the drug concentration in some parts of the body as compared to others [1]. The ultimate goal of any delivery system is to extend, confine and target the drug in the diseased tissue with a protected interaction. Every Dosage form is a combination of drug/active pharmaceutical ingredients (APIs) and the non-drug component called excipients/additives (Figure 1). APIs are the actual chemical components used to treat diseases [2].

### 1.1. Need for a Dosage Form

Generally, drug delivery systems (DDS) are preferred because direct clinical use of the active drug substances (APIs) “as they are” is very rare due to several reasons: API handling and accurate dosing can be difficult or impossible for very potent drugs (e.g., low mg and µg doses) [3]. Administration of drugs into the body cavities (rectal, vaginal) can be impractical and unfeasible as they can be degraded at the site of administration (e.g., low pH in the stomach) and may cause local irritations or injury when the drug concentration is high at the site of administration [3]. Some APIs are sensitive to the environment and can benefit from reducing the exposure to environmental factors (light, moisture, temperature and pH), or they need to be chemically stabilized due to the inherent chemical instability. APIs mostly have unpleasant organoleptic qualities (taste, smell and compliance), which reduce patient compliance [2].

Hence APIs are always formulated along with the excipients. Excipients/Additives are used: To give particular structure and shape to the formulation, to increase stability, to mask the bitter taste and increase palatability, to bulk up formulations that contain very potent active ingredients, to allow for convenient and accurate dosage, to aid in the handling of the active substance and to aid the manufacturing process [4]. In addition, excipients enhance the bioavailability, improve the overall safety or function of the dosage form during storage or in use with enhanced patient acceptability [5].

### 1.2. Excipients

One or more of the excipients that are generally utilized in formulations include: colouring agents, suspending agents, binding agents, solvents and lubricants, perfumes, sweetening agents, flavouring agents, solubilizing agents and antioxidants [4]. A filler is included to increase the size of the tablet (e.g., lactose) as often the amount of "active ingredient" is so small that the dosage form would be too tiny to handle without filler. Binders are added to hold the tablet together after it has been compressed, and prevent the break-down into separate pieces (e.g., starch, HPMC, etc.) [6]. Disintegrants help the dosage form to break down into small fragments after ingestion, which allows the medicine to dissolve and be absorbed by the body so that it can act more rapidly [6]. The glidants prevent lump formation by reducing the friction between particles and improve the flowability of the tablet granules or powder. Anti-adherents stop the powder from sticking to the machines during manufacturing. Lubricants ensure the smooth surface of dosage form, by reducing the friction between the walls of the tablets and the die cavity during ejection. Flavouring agents help to mask the unpleasant odour and colourants are added to aid in recognition and aesthetics [7].

### 1.3. Biopharmaceutics Classification System (BCS) Classification of Drugs

**Figure 2:**
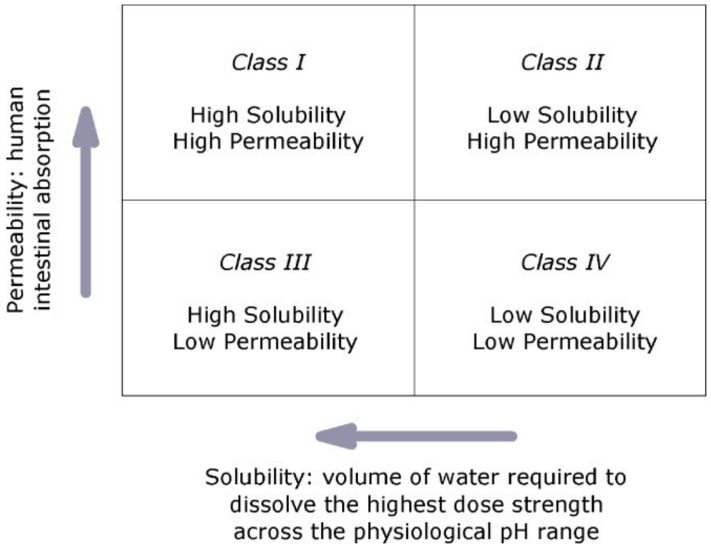
BCS Classification of drugs.

The Biopharmaceutics Classification System classifies drugs into four types based on their permeability (intestinal) and solubility (Figure 2) [8]. Class I drugs possess high permeability and high solubility, and are well absorbed; their absorption rate is greater than excretion (e.g., metoprolol, paracetamol, etc.). Class II drugs have high permeability but low solubility and the bioavailability is restricted by their rate of solvation (e.g., glibenclamide, aceclofenac, etc.) Class III drugs possess low permeability but high solubility where the drug solvates quickly; nevertheless, absorption is limited by the rate of permeation. If the formulation does not change the permeability or gastro-intestinal duration time, then class I criteria can be applied (e.g., cimetidine). Class IV drugs have low permeability and low solubility and are poorly absorbed through the intestine; thus, they have poor bioavailability with high variability (e.g., Bifonazole) [8].

### 1.4. Different Routes of Drug Administration

**Figure 3:**
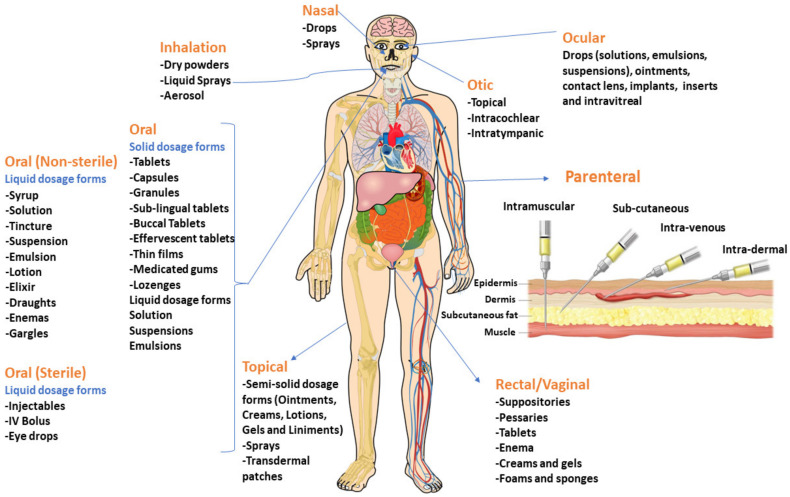
Various routes of drug administration.

Dosage forms can be administered through different routes based on the target site, duration of treatment and the physicochemical attributes of the drug [9]. The most common dosage forms comprise tablets, capsules, pills, ointments, syrups and injections. Various routes of drug administration are tabulated in Table 1 and Figure 3. The preferred route of drug administration depends on three main factors: The part of the body being treated, the way the drug works within the body and the solubility and permeability of the drug. For example, certain drugs are prone to destruction by stomach acids after oral administration resulting in poor bioavailability. Hence, they need to be given by the parenteral route instead. Intravenous administration of drugs gives 100% bioavailability [9].

## 2. Classification of Dosage Forms

**Figure 4:**
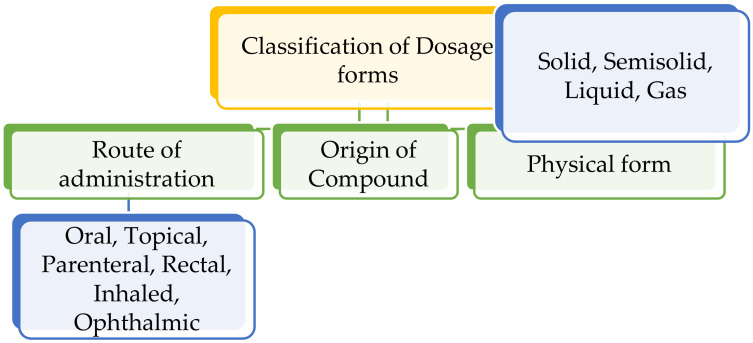
Classification of conventional dosage forms.

The dosage forms are classified based on the route of administration, the origin of the compound (natural/synthetic) and the physical form of the final delivery systems (Figure 4).

### 2.1. Classification of Solid Dosage Forms

**Figure 5:**
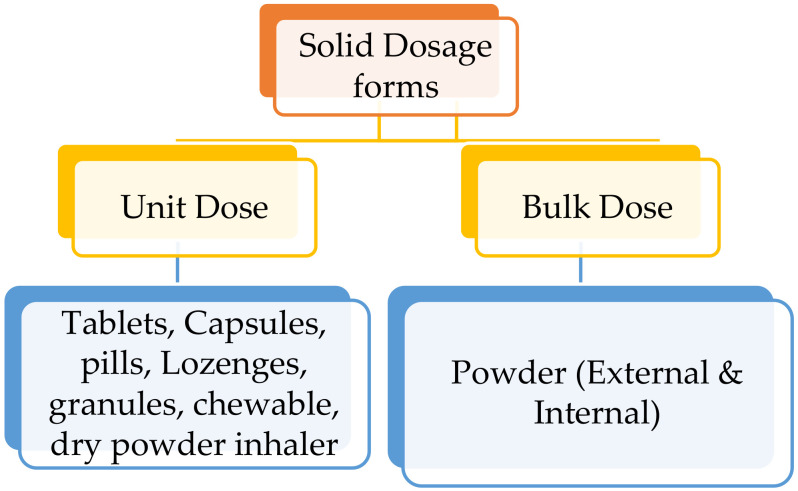
Classification of solid dosage forms.

Solid dosage forms are further classified into two main categories based on the type of dose, i.e., unit dose and bulk dose. (a) Unit dose: Each dose is fixed and formulated as a separate dosage form and the patient needs to take a single unit of a specific dose at a time. Examples of unit dosage forms include tablets, capsules, pills, lozenges, chewable tablets, effervescent tablets and dry powder inhalation in metered-dose containers. (b) Bulk dose: As the name itself says, it is a bulk solid powder where the individual dose is not formulated (Figure 5) [10,11]. Dose dumping is a major problem with bulk powders. However, bulk powders are generally used as dressing powder for surgical and injury wounds. Examples of bulk dosage forms include insufflation powder, dressing powder, etc. [10].

#### 2.1.1. Tablets

A tablet is a solid unit dosage form that is manufactured by compression and wet/dry granulation into different shapes (round, oval or square shape). For efficient tabletting, binders, glidants and lubricants are often added as excipients. To enhance the easy breakdown of tablets in the digestive tract, disintegrants are added. The tablet coating with pigments, sweeteners and flavouring agents helps to mask the taste of other ingredients and makes the tablet smoother and easier to swallow. Tablet coating also offers environmental protection and extends the shelf life [10,12].

Sublingual and Buccal tablets are also solid unit dosage forms administered by placing them under the tongue and between the gum and cheek, respectively. Advantages of sublingual/buccal delivery systems include: The medications dissolve rapidly and are absorbed through the mucous membranes of the mouth into the systemic circulation. This avoids the acid and enzymatic environment of the stomach and the drug-metabolizing enzymes of the liver [10,12].

Effervescent tablets are designed to evolve carbon dioxide when in contact with water and disintegrate within a few minutes. These are uncoated tablets consisting of acids (citric or tartaric acid) and carbonates or bicarbonates which react rapidly in water and release carbon dioxide. They are intended to be either dispersed or dissolved in water before intake to offer very rapid tablet dispersion and dissolution and release of the drug. It tastes similar to a carbonated drink (e.g., antacids). Chewable tablets are chewed before swallowing. They are designed for administration to deliver the drug by mastication. They are very useful for children and the elderly (e.g., vitamin products) [10,12].

#### 2.1.2. Capsules, Lozenges, Pills and Granules

**Figure 6:**
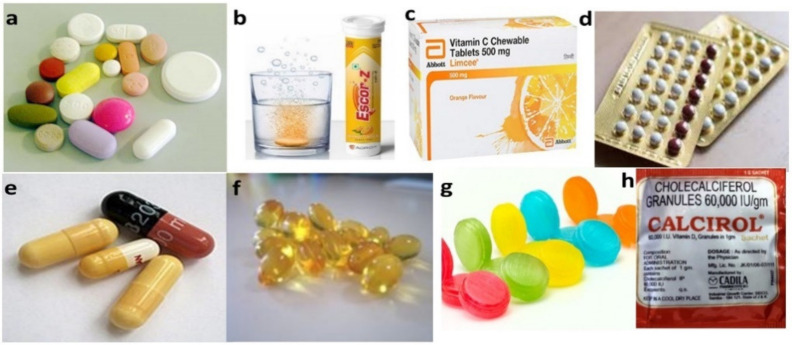
Solid unit dosage forms: (**a**) Tablets, (**b**) Effervescent tablets, (**c**) Chewable tablets, (**d**) Pills, (**e**) Hard-gelatine capsules, (**f**) Soft-gelatine capsules, (**g**) Lozenges. (**h**). Granules.

A capsule is a unit solid dosage form where the drug components are enclosed in a soluble shell. Capsules help to mask the unpleasant taste of its contents and the drug has limited interaction with the excipients. Capsules are classified into two types: Hard-shelled capsules, which are used to encapsulate dry, powdered components; soft-shelled capsules, principally used for hydrophobic drugs and oily active substances that are suspended or dissolved in oil. Lozenges are chewable solid unit dosage forms, where the drug is loaded in a caramel base made up of sugar and gum; the latter provides cohesiveness and strength to the lozenge and enables slow release of the drug. Lozenges are traditionally used for local slow release of demulcents, anaesthetics and cough remedies in the mouth/pharynx. Pills are solid unit dosage forms made by compressing API with adhesives and other excipients into rounded masses for oral administration. Granules are solid, dry aggregates provided as a single-dose in sachets which can either be placed on the tongue and consumed with water or dissolved in water before taking (Figure 6h). Effervescent granules evolve carbon dioxide similar to effervescent tablets when added to water. Figure 6 represents the examples of solid unit dosage forms [10].

#### 2.1.3. Bulk Solid Dosage Forms

Bulk Powders are multidose formulations comprising loose, solid and dry particles of variable fineness. One or more active ingredients are present with or without excipients and, if needed, colouring and flavouring agents are added. These are packed in wide-mouthed, air-tight, bulk containers made of glass or plastic, and are intended for either internal or external administration. There are two kinds of bulk powders intended for internal use.

Bulk powders are often limited by inaccurate dosage, since the patient measures each dose varyingly. Hence they are usually formulated with non-potent drugs such as laxatives, antacids, purgatives, etc., The powder is then typically dispersed in water or dissolved before taking. Divided powders are single-dose of powder (for example, a small sachet) with more accurate control on dosage than bulk powder [10].

### 2.2. Semisolid Dosage Forms

**Figure 7:**
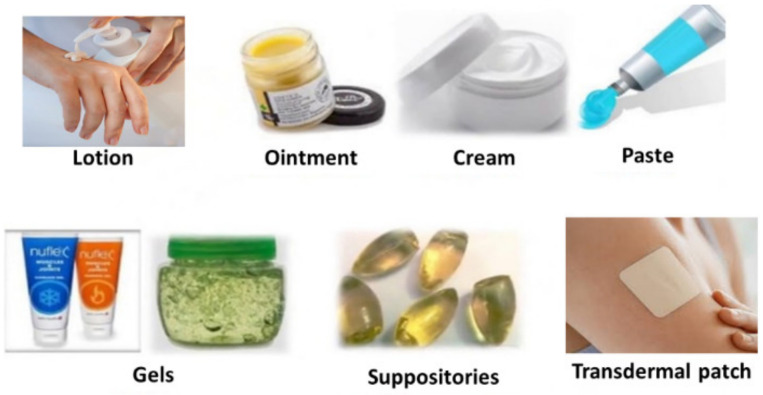
Semisolid dosage forms.

Semisolid dosage forms are of semisolid consistency intended to apply onto skin/mucous membranes (nasal, vaginal or rectal cavities) for therapeutic, protective or cosmetic applications. Semisolid dosage forms include ointments, creams, gel/jelly, lotions, pastes, suppositories and transdermal patches (Figure 7 and Table 2) [13]. Semisolid dosage forms are used externally and locally at the target site, which reduces the probability of side effects. It is convenient for unconscious patients or patients who have difficulty in oral administration. It is a suitable dosage form for bitter drugs and more stable than liquid dosage forms [14].

#### 2.2.1. Ointments

Ointments are oil-based semisolid formulations where the base is usually anhydrous and immiscible with skin secretions. These are made of less than 20% water and volatile substances, and more than 50% of hydrocarbons (waxes, or polyols) as the vehicle, due to which retention time for ointments is high and spread ability is less. Hence, ointments may be used as emollients or to apply suspended or soluble drugs to the smaller portions of skin for a longer duration [14,15].

#### 2.2.2. Creams

Creams are relatively soft, easy to spread, semisolid dosage forms which often contain more than 20% water and volatile substances and less than 50% hydrocarbons (waxes or polyols) as the base for the drugs. Cream bases are emulsions that are classified into two types: Oil-in-water (O/W) creams and water-in-oil (W/O) creams. Oil-in-water (O/W) creams are comprised of small oil globules dispersed in a continuous aqueous phase stabilized by surfactants [15]. Oil-in-water creams are more cosmetically tolerable as they are less greasy and simply washed off using water. Water-in-oil (W/O) creams are comprised of small droplets of water dispersed in a continuous oily phase. Hydrophobic drugs can easily be incorporated into W/O creams and, are also more moisturizing than O/W creams as they offer an oily barrier to prevent moisture loss from the outermost layer of the skin, the stratum corneum [14].

#### 2.2.3. Gels (Jellies) and Lotions

Gels are semisolid systems in which the liquid phase is confined in a 3D polymeric matrix (made up of natural or synthetic gums) with a high degree of physical or chemical cross-linking [16]. They are used in medicine, in cosmetics, for lubricating purposes and also as a drug carrier for spermicides used in the vagina [14]. A lotion is an aqueous fluid preparation for external use without friction. They are applied to the skin directly or pored on a suitable dressing and covered with a waterproof dressing to reduce evaporation [14].

#### 2.2.4. Pastes

A paste is basically an ointment with a high percentage of insoluble solids added. A large amount of particulate matter stiffens the system. As compared to the ointment, paste has lower permeability, lower maceration and lower heat. When applied to the skin, they form a good protective barrier [15]. The solids they contain can absorb and therefore neutralize certain harmful chemicals before they reach the skin. Like the ointment, the paste forms a complete film that is relatively impermeable to water [16]. Unlike the ointment, the film is opaque, so it can be used as an effective sunscreen. Since the fluid hydrocarbon fraction is absorbed by the particles, the paste is less greasy [14].

#### 2.2.5. Transdermal Patches

A transdermal patch or skin patch is an adhesive drug patch that is placed on the skin to deliver a specific dose of drug into the blood through the skin. For patients who are unable to take oral dosage forms or oral medications that cause intolerable side effects, the use of transdermal patches is strongly recommended as a treatment option [17]. However, this is not an appropriate method to control acute pain or clinical situations that require rapid titration of the drug. The transdermal patch is made up of a backing film, which is the outermost layer of the patch and provides protection for the drug components. The second layer consists of a drug contained in a film or adhesive. The membrane is a thin film that controls the diffusion rate of the drug from the patch to the skin. The adhesive layer helps the patch adhere to the skin [18]. As a functional layer or outer lining, the film-coated tape is directly integrated into the patch design. The release liner protects the sticky side of the patch which is going to be in contact with the skin and is removed before applying the patch to the skin [19].

**Figure 8:**
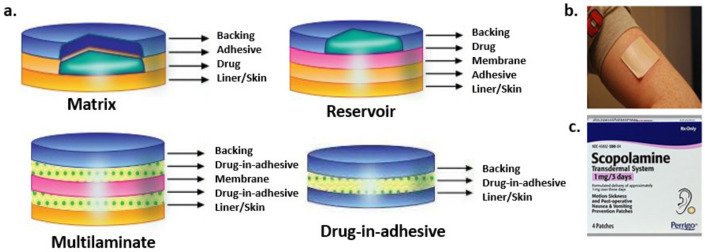
(**a**) Types of transdermal patches, (**b**) Transdermal patch applied on skin, (**c**) First commercially available Scopolamine transdermal patch (reproduced from [21] with permission from Perrigo, licensed under Creative Commons Attribution (CC BY 4.0) license).

Transdermal patches are classified into four types based on the drug loading type: Matrix, reservoir, multilaminate and drug-in-adhesive. The first type is a single-layer/multi-layer drug-in-adhesive transdermal patch, in which the drug is directly incorporated into the adhesive; the second type has a separate drug-containing layer, which is considered to be a drug reservoir; the third, called matrix transdermal patches, have a drug layer comprising a semisolid matrix containing a drug solution or suspension; and the fourth one is multilaminate having different layers of drugs (Figure 8). The molecular weight of the drug should be less than 500 Daltons to formulate as a transdermal patch. The drug should be sufficiently lipophilic for easy permeation through the skin. The dosage of the drug depends on the duration for which the patch is worn. The first commercially available patch was scopolamine for motion sickness [20].

#### 2.2.6. Suppositories

A suppository is a small, round or cone-shaped semisolid dosage form that is inserted into a body orifice (rectum, vagina) where it dissolves or melts to release the drug and exert local or systemic therapeutic effects. Suppositories are made up of natural fat (cocoa butter) or polyethylene glycol (Carbowax) and glycerol as main excipients. They are exclusively intended to be introduced in the anus and show a rapid onset of action since the rectum is highly vascularized; besides, they bypass the hepatic first-pass metabolism [14,22].

### 2.3. Liquid Dosage Forms

**Figure 9:**
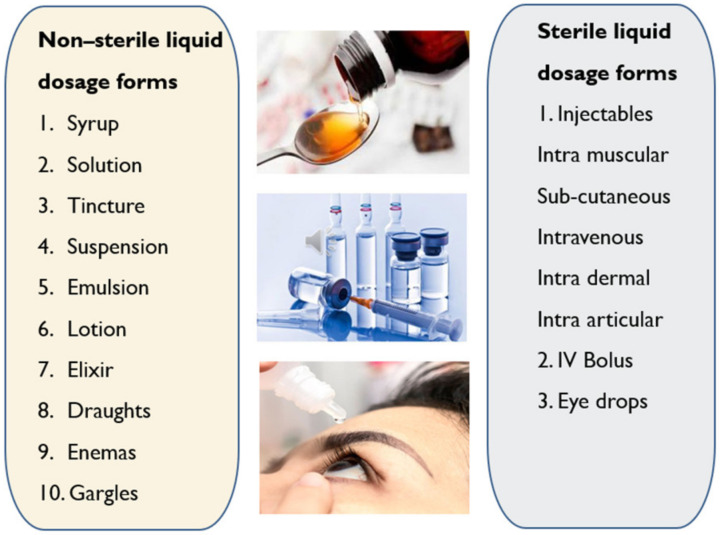
Sterile and non-sterile liquid dosage forms.

Liquid dosage forms are pourable pharmaceutical formulations comprising of API and excipients either dissolved or dispersed in a suitable solvent/s. These are intended to offer a fast therapeutic response in people with trouble swallowing solid dosage forms. Liquid dosage forms are available as ready-to-use liquids or dry powders for reconstitution. These can be administered by oral (syrups, suspensions, etc.) and/or parenteral (injectable, ophthalmic, nasal, otic and topical) routes. Oral liquids are generally nonsterile, while the parenteral liquid dosage forms are offered as sterile and non-sterile formulations (Figure 9). Liquid dosage forms are classified based on the number of phases present into two types: Monophasic (solutions) and biphasic (suspensions and emulsions) [23].

- Oral solutions are monophasic clear liquids for oral use comprising of one or more active ingredients dissolved in a suitable solvent system [24].
- Oral emulsions are biphasic liquids for oral use where the drug is present in oil-in-water emulsion either in single or dual phases [25].
- Oral suspensions are biphasic liquid dosage forms for oral use comprising of one or more APIs suspended in a suitable solvent. They tend to sediment with time; nevertheless, they can be readily re-dispersed by shaking into a uniform suspension that remains appropriately stable to allow the accurate dose to be delivered [24].
- Syrup is a concentrated aqueous sugar solution, usually sucrose, in which APIs are dissolved. Flavoured syrups are suitable to mask the unpleasant taste of drugs [25].
- Elixir is monophasic clear liquids for oral use for administering potent or nauseous drugs by adding pleasant flavours. The vehicle comprises a high amount of ethanol or sucrose along with antimicrobial preservatives to enhance the stability of the formulation [25].
- Linctuses are viscous oral liquids made of a high amount of syrup and glycerol which have a demulcent effect on the membranes of the throat and are used for cough relief. These are taken in smaller doses (<5 ml) and undiluted to prolong the demulcent action [26].
- Oral drops are either solutions, suspensions or emulsions that are administered in very small volumes (<1 ml) into the eyes, nose or ears [27].
- Gargles are concentrated aqueous solutions that need to be diluted with warm water before use to wash the mouth and throat by holding the liquid in the throat and agitate it by the air from the lungs [28].
- Mouthwashes are similar to gargles but are used to maintain food oral hygiene and also to prevent infections in the mouth [23,28].

## 3. Pharmacokinetics of Drug Delivery Systems

**Figure 10:**
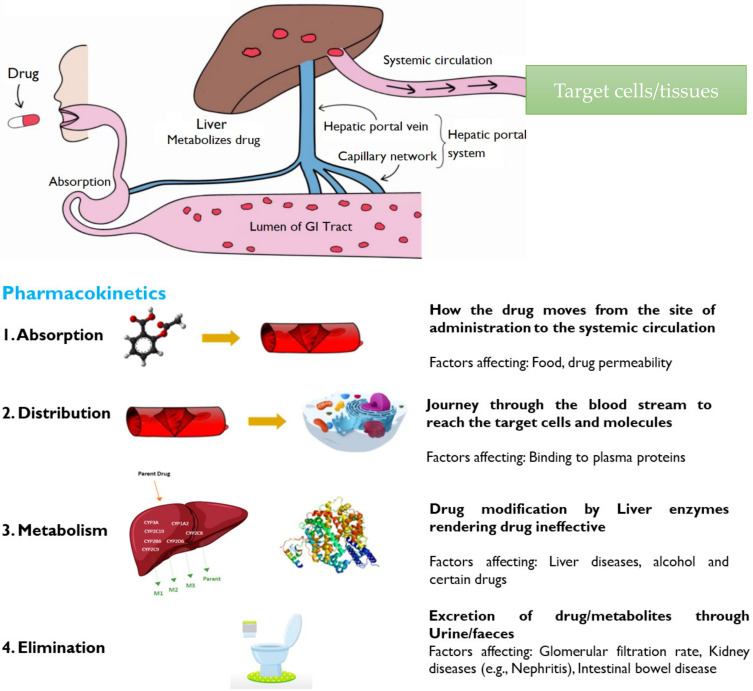
Pharmacokinetic phases of a drug: 1. Absorption, 2. Distribution, 3. Metabolism, 4. Excretion.

*Pharmacokinetics* is the movement of drugs into, through and out of the body—the time course of drug absorption, distribution, metabolism, and excretion. In simple terms, it is what the body does to a drug [29]. A schematic illustration of pharmacokinetics is represented in Figure 10.

### 3.1. Absorption

Absorption is the movement of a drug from its site of administration to the bloodstream. The rate and extent of drug absorption depend on several factors, such as route of administration, physicochemical properties of the drug, type of formulation and drug–food interactions [30,31]. The fraction or amount of drug (in active form) that reaches the target site through the systemic circulation is called bioavailability. Intravenous administration of the drug offers 100% bioavailability as the dosage form is directly administered into the bloodstream. Oral dosage forms suffer from poor bioavailability due to incomplete absorption and hepatic first-pass effect which metabolizes the drug in the liver, rendering it less active or inactive. Absorption of the drug through the plasma membrane occurs by either passive transport or active transport [30].

**Figure 11:**
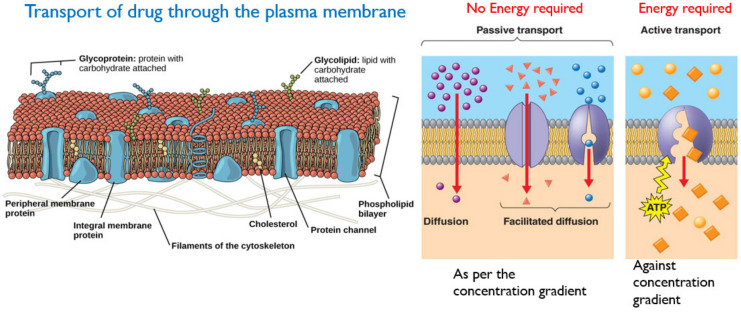
Schematic of transport of drug through the plasma membrane by passive transport and active transport (reproduced from [34] with permission from the OpenStax, (part of Rice University, which is a 501(c)(3) nonprofit) and [35] licensed under Creative Commons Attribution (CC BY 4.0) international license).

(a) Passive Transport involves the movement of the drug across the cell membrane from the high drug concentration region (such as gastrointestinal tract), to the low drug concentration region (such as blood). This is a passive process and no energy is required, and the rate of drug diffusion is directly proportional to the concentration gradient [32]. Other factors influencing passive transport include the physicochemical properties of the drug, such as its lipid solubility, molecular size, degree of ionization and the absorptive surface area available to the drug [30].
(b) Active transport requires energy to facilitate the transport of drug molecules against a concentration gradient, which usually occurs at specific sites in the small intestine. The majority of drugs that are absorbed via active transport share a similar structure with endogenous substances such as ions, vitamins, sugars and amino acids [30,33]. A schematic of active and passive transport is given in Figure 11.

### 3.2. Distribution

**Figure 12:**
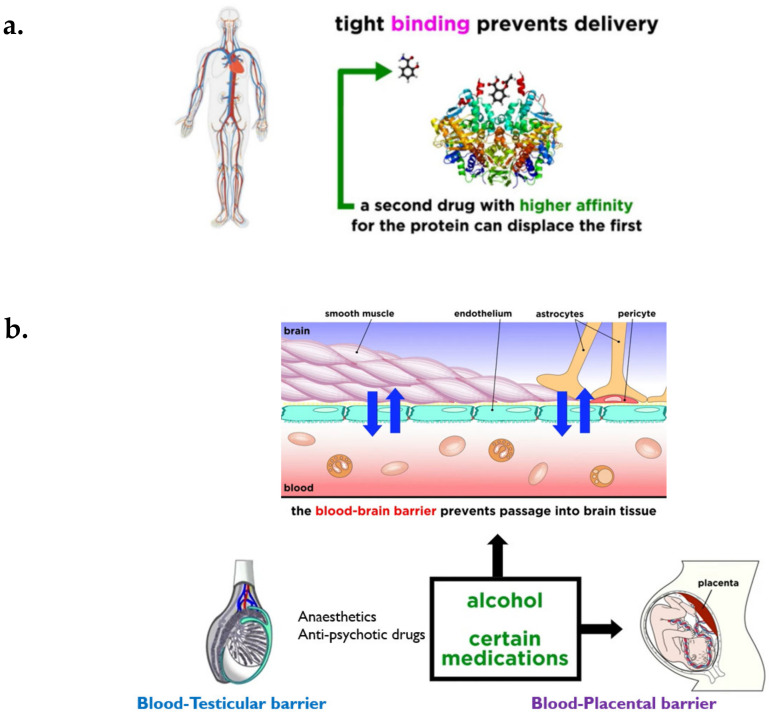
Schematic of barriers to drug distributions (**a**) Plasma protein binding, (**b**) Anatomical barriers.

Distribution is a reversible transfer of a drug between the blood and the extra vascular fluids and tissues of the body (for example fat, muscle, and brain tissue). Drug distribution governs the amount of drug reaching target sites as compared to the rest of the body, and thus plays an important role in drug efficacy and toxicity. Various factors affecting drug distribution include blood flow, lipophilicity and molecular size of the drug, and binding affinity of the drug with plasma proteins [36,37]. For example, a drug with a high protein-binding affinity (e.g., warfarin), possesses a very little amount of free drug in the target site to exert a desired therapeutic response. Warfarin drug, due to strong protein binding efficacy, can replace any other drug bound to plasma proteins and allow it to be free to show the therapeutic response [30]. Additionally, there are anatomical barriers found in certain organs like the blood–brain barrier, preventing certain drugs from going into brain tissue (Figure 12). Drugs with high lipophilicity, smaller size, and low molecular weight can cross the blood–brain barrier [29].

### 3.3. Metabolism

**Figure 13:**
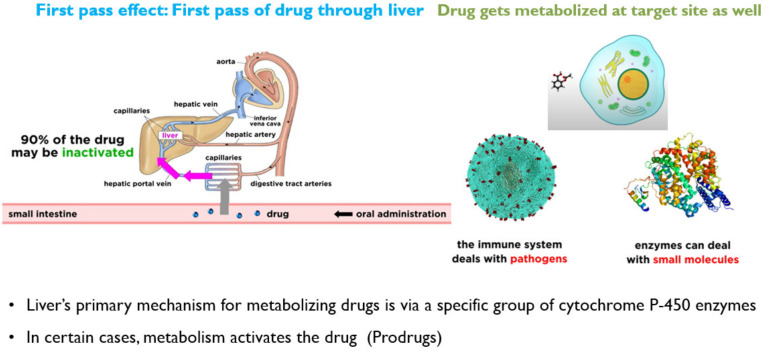
Schematic of drug metabolism in the liver as well as the cells.

The metabolism of drugs (in the gut wall and liver) into inactive or less active components before being absorbed into the systemic circulation. The concentration of a drug, especially after oral administration, is significantly reduced before reaching the bloodstream [37,38]. It is the fraction of drug that is lost during absorption, and cytochrome P450 (CYP450) enzymes of the liver are accountable for the metabolism or biotransformation of about 70–80% of the drugs in clinical use [30]. The drug metabolism is schematically explained in Figure 13.

### 3.4. Excretion

**Figure 14:**
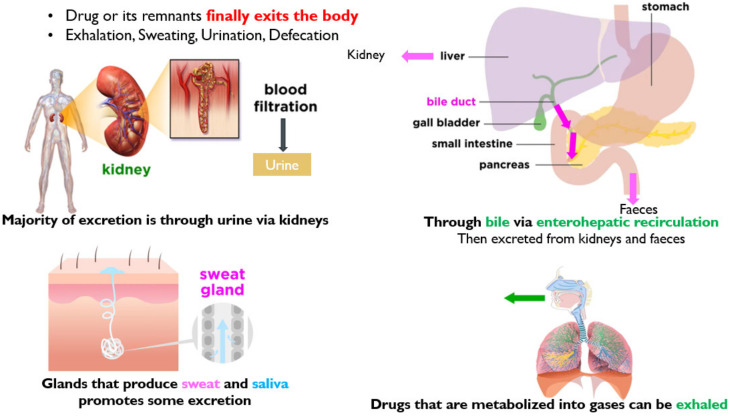
Schematic illustration of drug excretion from the body by kidneys, liver, skin and airways.

The removal of unchanged drugs or their metabolites from the body is called drug excretion [39]. There are many different routes of excretion, including urine, bile, sweat, saliva, tears, milk and stool [30]. An illustration of various modes of drug excretion is presented in Figure 14.

### 3.5. Bioavailability

**Figure 15:**
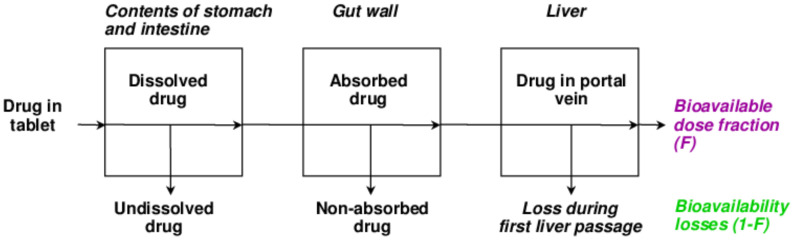
Schematic of factors accountable for the reduction in bioavailability.

This is the fraction or percentage of administered drug absorbed into the systemic circulation. Drugs with high hepatic metabolism and faster excretion have low bioavailability. The sub-therapeutic dose is present at the target site and results in low efficacy. Hence, for low bioavailable drugs, high dosage is needed. Drugs that are absorbed via the Gastro-Intestinal Tract (GIT) are circulated to the liver first via the hepatic portal vein. The liver then acts as a filter (CYP enzymes metabolize). Only part of the drug is reached systemically. The greater the first pass effect, the lesser the bioavailability. The IV route offers 100% bioavailability [40,41]. A schematic of factors accountable for the reduction in bioavailability is represented in Figure 15.

### 3.6. Biological Half-Life (t_1/2_)

Elimination half-life or Biological half-life (t_1/2_) is the time at which the mass of an unchanged drug becomes half (50%) of the initial concentration. Simply, t_1/2_ refers to how long it takes for half of the administered dose to be metabolized and eliminated from the bloodstream [42]. The half-life of a drug can be determined using the following equations:

**t_1/2_ = (0.7 × V_d_)/Cl**, where V_d_ is volume of distribution and Cl is clearance.

**t_1/2_ = 0.693/K_t_**, where K_t_ is the Elimination rate constant.

Drugs with a short biological half-life need frequent dosing to achieve a therapeutic response for a longer duration. The goal is to maintain the therapeutic blood level over extended periods, for which the drug must enter the systemic circulation approximately at the same rate at which it is eliminated. Elimination of a drug varies due to factors like age, weight, other medications taken, other medical conditions present, kidney function, liver function, etc. Therefore, the half-life is used as a guide or an estimate of how long it may take for the drug to be removed from the body [41].

## 4. Drug Release Kinetics Basic Concepts

**Figure 16:**
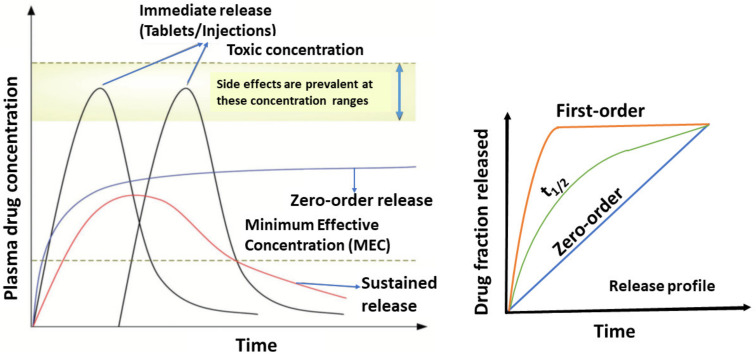
Drug plasma levels and release profiles.

The drug release profile is generally expressed as a plot of plasma-drug concentration versus time. In the plot shown in Figure 16, two important concentration levels are shown: The minimum effective concentration, below which the drug is ineffective, and the toxic concentration, above which undesirable side effects occur. Maintenance of drug concentration at any instance between minimum effective concentration to minimum toxic concentration is critical for safety and therapeutic effectiveness [42]. Drug release kinetics is said to be zero-order kinetics when a constant amount of drug is eliminated per unit time but the rate is independent of the concentration of the drug. Zero-order DDS have the potential to overcome the issues faced by immediate-release and first-order systems by releasing the drug at a constant rate, thereby maintaining drug concentrations within the therapeutic window for an extended period [43,44].

Minimum effective concentration (MEC): The lowest level of concentration of drug in the body that shows desired therapeutic effect [45].

Zero-order release: Zero-order kinetics is described when a constant amount of drug is eliminated per unit time but the rate is independent of the concentration of the drug [45].

First-order release: The drug release rate is directly proportional to the concentration gradient and is a function of the amount of drug remaining in the dosage form [45].

Sustained release: This is designed to achieve slow release of a drug over an extended period after administration of a single dose [45].

### Therapeutic Index (TI) and Therapeutic Window

**Figure 17:**
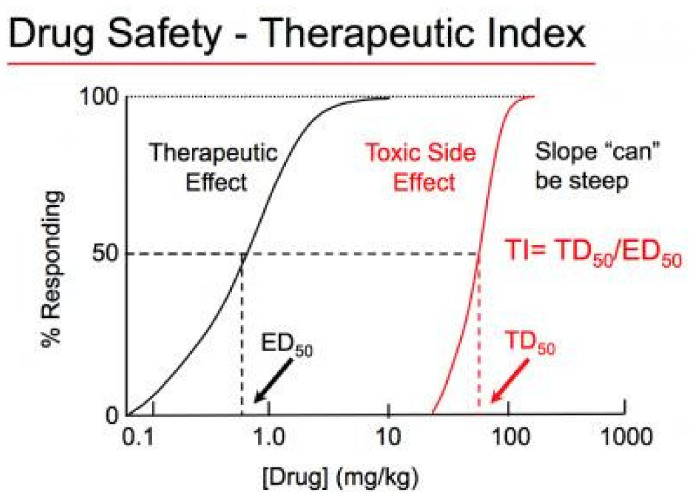
Schematic of drug safety-therapeutic index.

The therapeutic index (TI; also referred to as therapeutic ratio) is a quantitative measurement of the relative safety of a drug. It is a comparison of the amount of a therapeutic agent that causes the therapeutic effect to the amount that causes toxicity. A therapeutic window or safety window refers to the range of doses that optimize between efficacy and toxicity, achieving the greatest therapeutic benefit without resulting in unacceptable side effects or toxicity [45]. TI is calculated from the ratio of the dose of a drug that causes adverse effects at an incidence/severity not compatible with the targeted indication (e.g., toxic dose in 50% of subjects, TD_50_) to the dose that leads to the desired pharmacological effect (e.g., efficacious dose in 50% of subjects, ED_50_) (Figure 17) [46].

## 5. Conventional vs. Controlled Drug Delivery Systems

**Figure 18:**
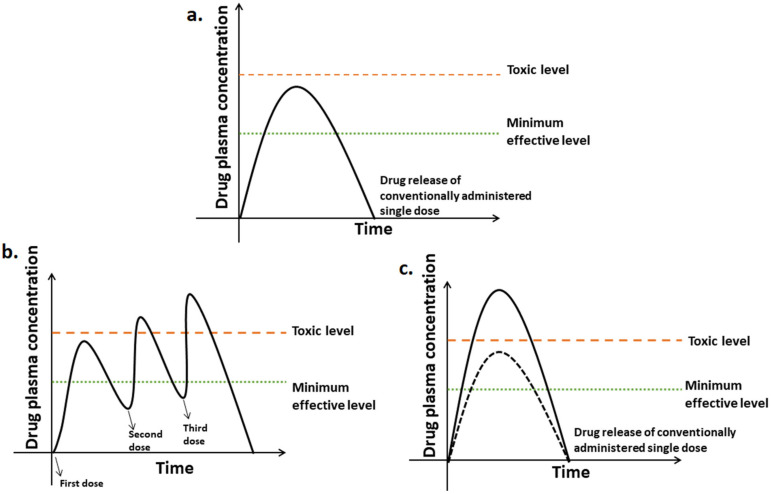
Plasma drug levels with time after administering (**a**) Single conventional dose, (**b**) Multiple doses, (**c**) Increased single dose.

**Figure 19:**
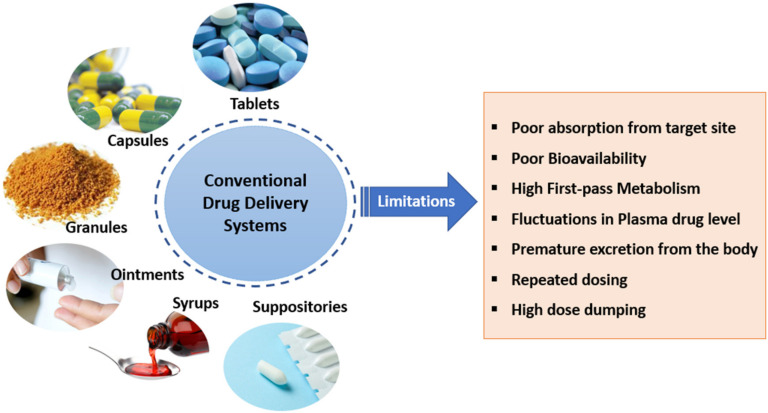
Limitations of Conventional drug delivery systems.

Conventional DDS (tablets, capsules, syrups, etc.) get eliminated from the body very quickly and the dose is not well maintained within the therapeutic window. After taking a single conventional dose, the drug metabolizes very quickly and the drug level increases, immediately followed by an exponential decrease. The time frame may not be long enough to produce a significant therapeutic effect and result in a sub-therapeutic response. Figure 18 illustrates the plasma drug fluctuations in conventional DDS. Hence, to maintain the plasma drug concentration above the minimum effective concentration (MEC) and below the toxic concentration, multiple approaches have been sought. Administering multiple doses at regular intervals of time might seem to be an alternative to a single dose, but the former results in fluctuations in plasma drug levels and often reaches below effective levels and above toxic levels. Taking several doses within a day result in poor patient compliance. Another approach is by administering a single dose greater than the required dose, which leads to adverse effects other than the effects intended by the drug (Figure 18). Hence, controlled release DDS are required to maintain the plasma drug levels at a constant rate within the therapeutic window and offer the desired therapeutic effect for a longer duration of time. [43]. A schematic of the disadvantages of conventional DDS is given in Figure 19. The advantages and disadvantages of conventional and controlled DDS are presented in Table 3 and Table 4.

### Sustained Drug Delivery System

Sustained-release drug delivery systems achieve the slow release of a drug over an extended period after administration of a single dose.

## 6. Controlled Drug Delivery Systems

**Figure 20:**
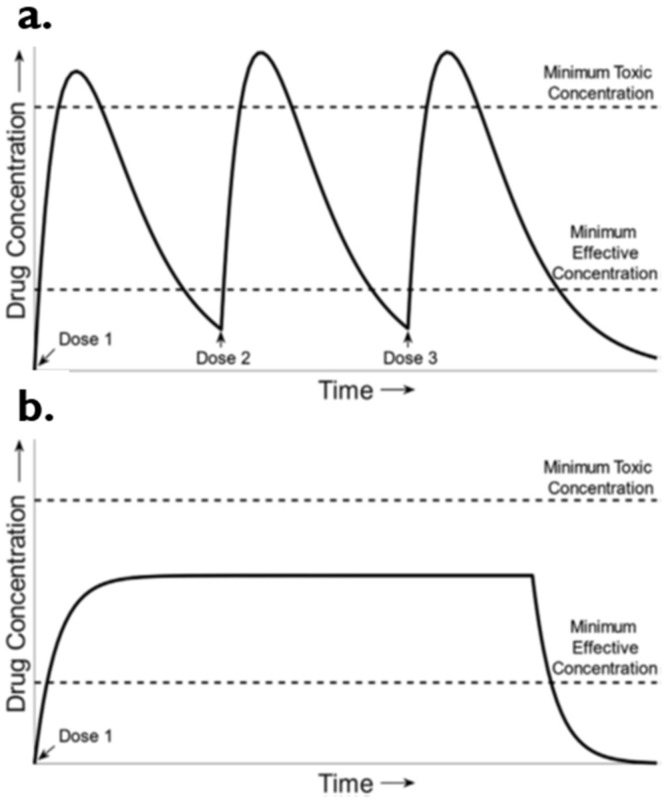
A typical bolus of (**a**) Conventional DDS; (**b**) Controlled DDS.

This is the drug delivery system in which a constant level of a drug is maintained in blood and tissue for an extended period. Pharmacokinetics (PK) curves of plasma concentration of a drug versus time for two types of delivery systems, conventional and controlled, are represented in Figure 20. In a conventional delivery system, there is typical bolus PK for multiple dosing with oral tablets or injections, where the drug level fluctuates above and below the minimum effective concentration. The controlled delivery system, on the other hand, shows zero-order PK with just a single dose of controlled drug delivery from a specific formulation or device. The drug levels are maintained constantly within the therapeutic window [47].

**Figure 21:**
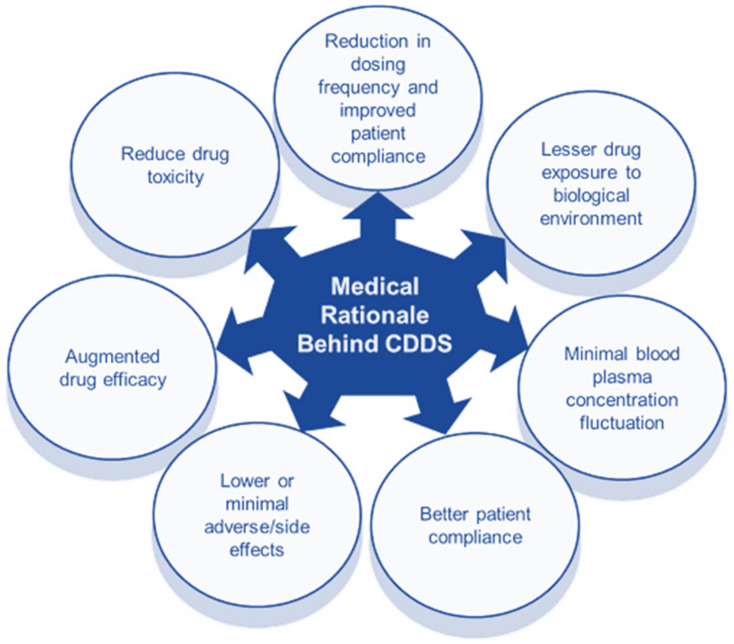
Medical Rationale behind controlled release drug delivery systems (CRDDS).

Controlled DDS maintain drug plasma levels constantly by releasing the definite dose of the drug at each time point for a pre-determined duration. This helps in reducing the dose and dosing frequency and improves patient compliance. Lesser drug exposure to the biological environment reduces drug toxicity and adverse effects. The overall efficacy of the dosage form is augmented [43]. The medical rationale behind controlled DDS is schematically represented in Figure 21.

### 6.1. Design Considerations of Controlled Release Drug Delivery Systems

**Figure 22:**
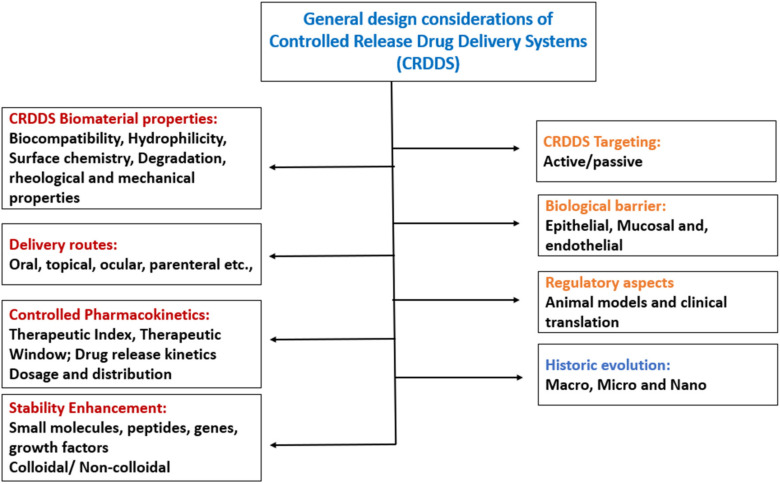
General design considerations of controlled release drug delivery systems (CRDDSs).

In designing a controlled release drug delivery system, various factors and parameters need to be considered; Figure 22 briefly illustrated the design considerations. The parameters are broadly classified as formulation related and drug related. Under formulation-related parameters, the biomaterial properties, route of administration, pharmacokinetics and stability enhancement are the major factors. In addition, the drug-related parameters include drug binding efficiency with plasma proteins and the ability of the drug to cross biological barriers and regulatory aspects are also the foremost criteria in designing the dosage form [43].

Biomaterial properties such as biocompatibility, surface chemistry, hydrophilicity, degradation, mechanical and rheological properties need to be studied. In addition, the behaviour of the biomaterials at various pH and temperatures also needs to be assessed. The routes of drug administration are critical for choosing the suitable biomaterial and designing the dosage form. For instance, rectal administration needs the melting point of the biomaterial to be at or above 37 °C or it is soluble at that pH so that the drug gets released. For certain drugs which are not stable in harsh conditions, including peptides, proteins, genes (DNA), growth factors and colloidal/non-colloidal particles, the stability enhancement should be done while designing the controlled release carrier. This can be achieved by incorporating the particular drugs in specialized carrier systems [48].

Targeting the drug to the site wherever the intended pharmacological activity is needed is of utmost importance to prevent the unwanted drug effects on other organs. This could be achieved by antibody tagging, attaching ligands and localized delivery. The biological barriers are a hindrance to targeting drug delivery to certain areas including the brain, bone and testicles. Drugs formulated with permeation enhancers and nanocarriers are the alternatives that can cross the barriers and deliver the drug to the target site [49]. Suitable animal models need to be established for each kind of delivery system to get the best in vitro in vivo co-relationship (IVIVC). This helps to bridge the gap between in vivo animal studies and the clinical study results [50].

### 6.2. Classification of Controlled Release Drug Delivery Systems

Controlled release drug delivery systems are classified based on the mechanism of drug release from the dosage form into dissolution controlled, diffusion-controlled, water penetration-controlled (osmotic pressure-controlled and swelling-controlled), chemically controlled and nanoparticle-based systems [51].

#### 6.2.1. Dissolution Controlled Drug Delivery Systems

**Figure 23:**
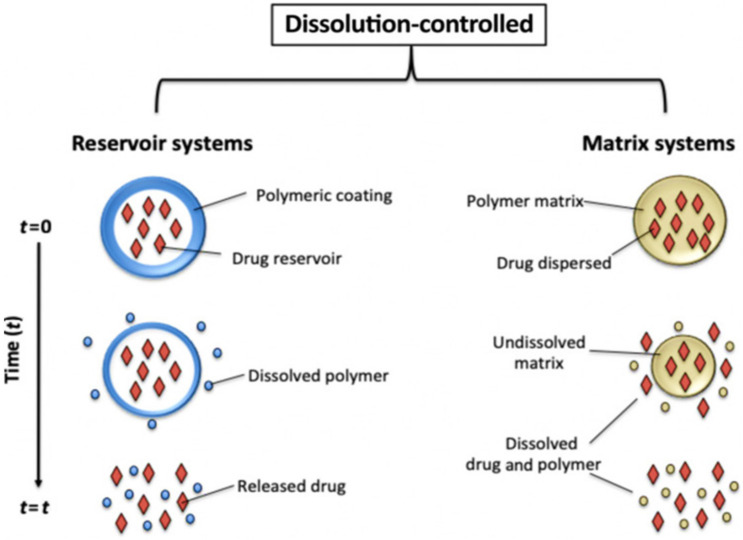
Dissolution-controlled delivery systems.

In dissolution-controlled release systems, drugs are either coated with or encapsulated within slowly dissolving polymeric membranes (reservoir systems) or matrices (monolithic systems), respectively (Figure 23). In reservoir systems, drugs are protected inside polymeric membranes with low solubility. Most of the conventional immediate-release tablets, pills and effervescent tablets are dissolution-controlled systems, where the rate-limiting step is dissolution [52].

#### 6.2.2. Diffusion-Controlled Drug Delivery Systems

**Figure 24:**
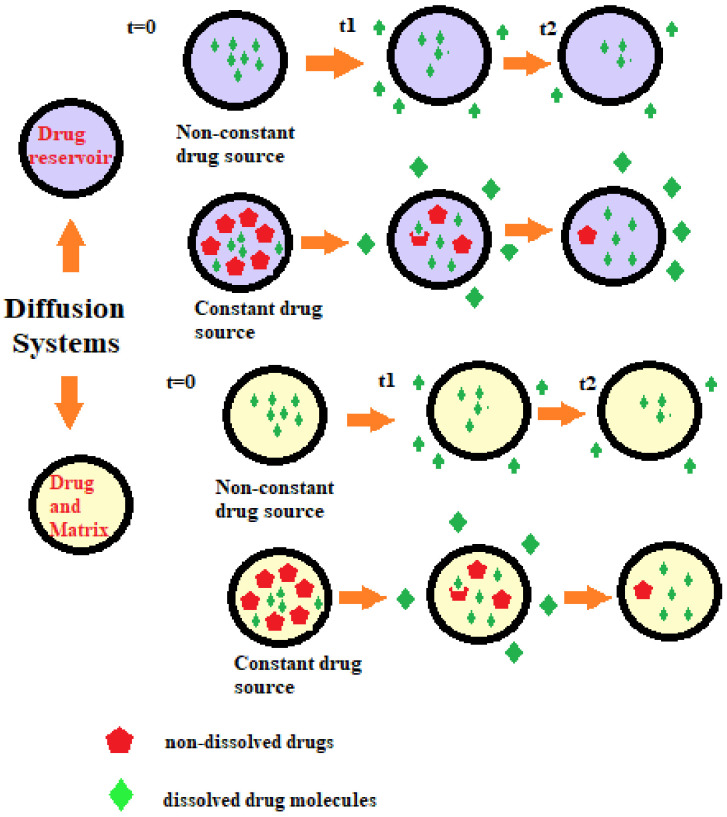
Schematic of diffusion-controlled delivery systems.

In diffusion-controlled release systems, drugs are trapped in and released via diffusion through inert water-insoluble polymeric membranes (reservoir systems) or polymeric matrices (monolithic systems). These are classified into membrane control reservoir systems and monolithic matrix systems (Figure 24). The drug release is governed by Fick’s laws of diffusion. The rate-limiting step in diffusion-controlled systems is the diffusion of drugs [53,54].

Fick’s first law of diffusion (Equation (1)) states that the molar flux (J) due to diffusion is proportional to the concentration gradient (*dc/dx*). Fick’s second law (Equation (2)) states that the rate of change of concentration of the solution at a point in space is proportional to the second derivative of concentration with space. It gives deals with changes in concentration gradient with time at any distance. The drug release which obeys Fick’s law is said to be Fickian diffusion, while those which do not obey are considered as non-Fickian or anomalous diffusion [53].

Fick’s first law:

(1) $$J\propto \;\frac{dc}{dx}\;\mathrm{or}\;J=D.\frac{dc}{dx}$$

Fick’s second law:

(2) $$\frac{dc}{dt}=D.\;\frac{d^{2}c}{dx^{2}}$$

dc = change in concentration of drug (g/cm^3^),

dx = change in distance (cm),

D = diffusion constant (cm^2^/s),

J = flux (cm^−2^ s^−1^),

dt = change in time (s).

**Figure 25:**
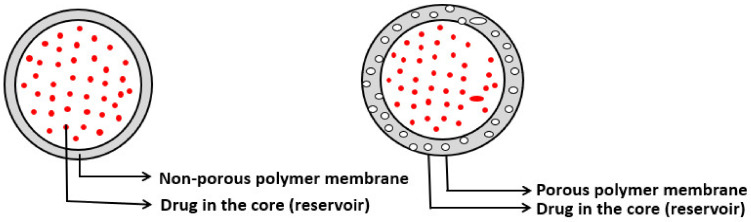
Membrane-controlled drug delivery systems.

Diffusion-controlled systems are classified into membrane-controlled and monolithic or matrix systems. In membrane-controlled systems, the drug is contained in the core as a reservoir and is covered by a thin polymeric membrane. The membrane could be either porous or non-porous. The release of drugs is by diffusion through the membrane and the rate of release is governed by membrane thickness, porosity and physicochemical characteristics of drugs (partition coefficient, molecular size and diffusivity, protein binding and dosage). Common methods to fabricate membrane-controlled reservoir systems include encapsulation and press coating of tablets [53]. A schematic of membrane-controlled delivery systems is given in Figure 25.

**Figure 26:**
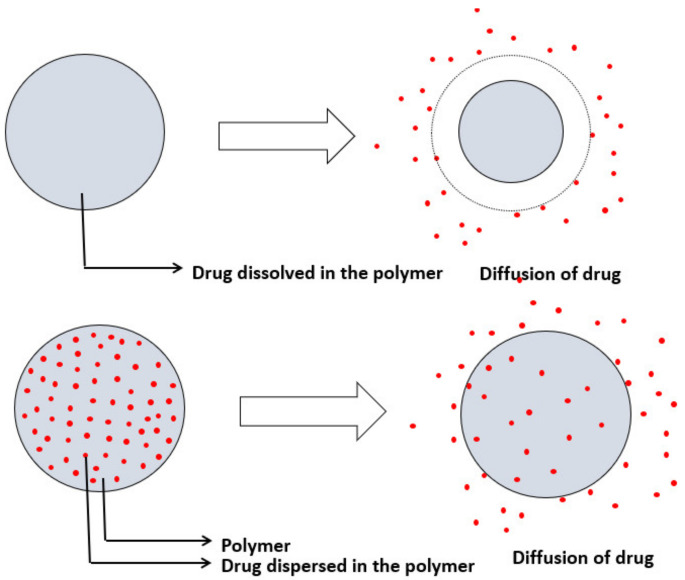
Monolithic/matrix-controlled drug delivery systems.

In monolithic or matrix-controlled delivery systems, the drug is either dissolved or dispersed homogenously throughout the polymer matrix. The drug release is through diffusion when the outside layer that is exposed to the solution gets dissolved first, allowing drugs to diffuse out of the matrix. In monolithic systems, where a drug is dissolved, drugs are loaded below the solubility limit. As the size of the matrix decreases, the drug released decreases. Here the drug release is nonzero order, i.e., rate of absorption ≠ rate of elimination. In monolithic systems where the drugs are dispersed in the polymer matrix, drugs are loaded above the solubility limit [53]. A schematic of monolithic-controlled delivery systems is given in Figure 26. Differences between monolithic and matrix systems are illustrated in Table 5.

#### 6.2.3. Water Penetration-Controlled Drug Delivery Systems

These are classified as osmotic pressure-controlled drug delivery systems and swelling controlled drug delivery systems. The rate control is dependent on water penetration into the system.

##### Osmotic Controlled Drug Delivery Systems

Osmotic drug delivery uses the osmotic pressure for controlled delivery of drugs by using osmogens. Osmosis refers to the process of movement of solvent from a lower concentration of solute towards a higher concentration of solute across the semipermeable membrane. Osmotic pressure is the pressure exerted by the flow of water through a semipermeable membrane separating two solutions with different concentrations of solute. These systems can be used for both routes of administration, i.e., oral and injectables [55].

**Figure 27:**
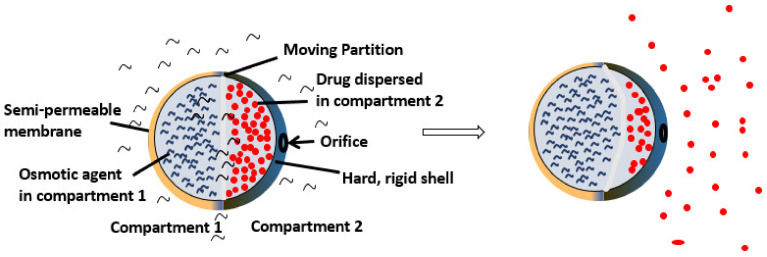
Schematic of osmotic pressure-controlled drug delivery system.

Basic components of osmotic DDS include the drug which itself may act as osmogen; otherwise, osmogenic salt can be added to the formulation. A semipermeable membrane with sufficient wet strength and water permeability that is biocompatible and rigid in withstanding the pressure within the device is needed. Apart from that, an outer coating material that is permeable to water but impermeable to solute can be used. Polymers such as cellulose acetate, cellulose triacetate and ethyl celluloses are commonly used in osmotic drug delivery systems. The advantages of osmotic-controlled delivery systems include increased efficacy of the drug, controlled drug delivery and reduced dosing frequency [56]. A simple osmotic delivery system is a pump that is made up of two compartments separated by a moving partition as shown in Figure 27. Compartment one is filled with an osmotic agent covered by a semi-permeable membrane. Compartment 2 is covered by a hard rigid shell with a delivery orifice [56].

**Figure 28:**
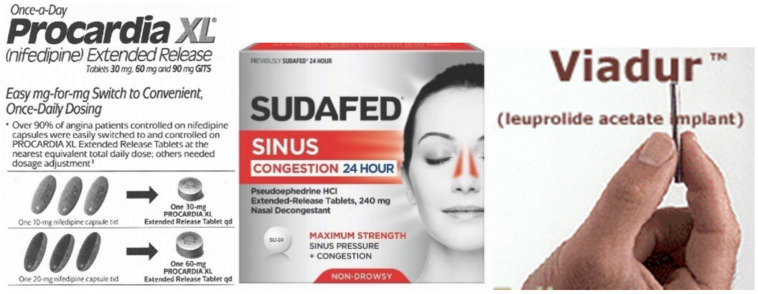
Example of osmotically controlled drug delivery systems (reproduced from [58,59,60] with permission from Pfizer Laboratories Div Pfizer Inc., Johnson & Johnson Consumer Inc. 2019 and Bayer licensed under Creative Commons Attribution (CC BY 4.0) license).

Examples for osmotic pressure-controlled drug delivery systems (Figure 28) in the market include: (a) Cardura^®^ XL (doxazosin mesylate) sold in Germany for the treatment of hypertension; (b) Covera-HS^®^ (verapamil), a controlled release system for the management of hypertension and angina pectoris; c) Sudafed^®^ (pseudoephedrine) for 24-h relief of cold and other respiratory allergies; d) Procardia XL^®^ (nifedipine) extended-release tablet for the treatment of angina and hypertension. Products incorporating ALZA’s DUROS^®^ Implant Technology Viadur^®^ (leuprolide acetate implant) deliver leuprolide continuously for 12 months [56,57].

##### Swelling-Controlled Drug Delivery Systems

**Figure 29:**
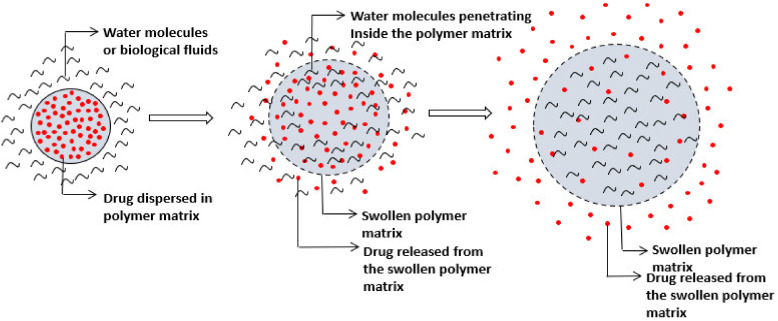
Schematic of mechanism of drug release from the swelling-controlled drug delivery systems.

In swelling-controlled drug delivery systems, the drug is dispersed or dissolved in the hydrophilic polymer when in a glassy (hard and rigid) state. In an aqueous solution, water penetrates the matrix and the glass transition temperature of the polymer is lowered below ambient temperature. This makes the matrix swollen and rubbery, which results in slow drug diffusion out of the swollen rubbery polymer matrix (Figure 29) [61].

#### 6.2.4. Chemically Controlled Drug Delivery Systems

**Figure 30:**
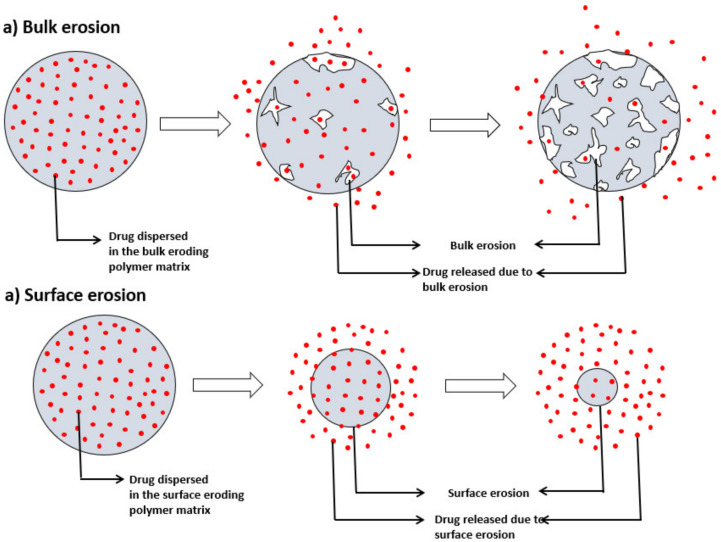
Schematic of bulk erosion and surface erosion.

Chemically controlled delivery systems change their chemical structure when exposed to the biological milieu. These are made of biodegradable polymers which degrade in the body as a result of natural biological processes, eliminating the need to remove the delivery system after exhausting an active agent from the system. These are classified into two types: Polymer-drug dispersion system and polymer-drug conjugate systems. In polymer-drug dispersion systems, the drug is uniformly dispersed or dissolved in a biodegradable polymer and released through degradation of polymers under physiological conditions. Two types of biodegradations are reported: bulk erosion, which is through breakdown of polymers in the bulk and, surface erosion which is due to the breakdown of polymers from the surface or dissolution of polymers from the surface (Figure 30). Various factors that affect degradation (bioerosion and bulk erosion) include chemical structure and composition, the presence of unexpected units or chain defects, configuration and molecular weight [62].

**Figure 31:**
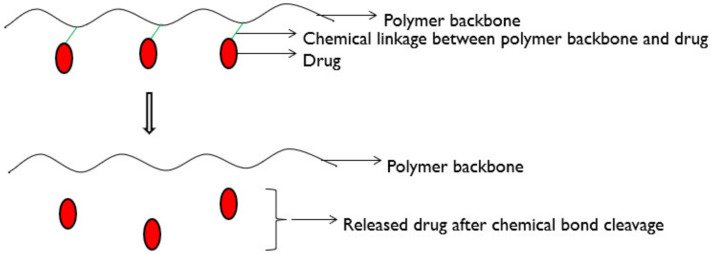
Polymer-drug conjugate systems.

In polymer-drug conjugate systems, the drug is chemically conjugated with the polymer either by covalent bonding or grafting to the backbone of the polymer. The drug releases through the cleavage of polymer-drug bonds under physiological conditions (Figure 31).

## 7. Controlled Release Dosage Form Design: Practical Considerations

The scientific rationale for the development of controlled drug delivery systems is to reduce the dose and frequency of dosage, reducing the fluctuations of blood plasma levels, patient compliance and adverse effects, and a reduction in the toxicity of the drug. The rate of availability of the drug in the body is maintained by the physiology of absorption in the immediate release system of the drug whereas, in the case of the controlled drug delivery, the rate of administration depends on the dosage of the drug. The main purpose of the controlled drug delivery is to minimise the frequency of drug administration. To achieve the required therapeutic concentration of the drug and to maintain the concentration of the drug for a specific time, the delivery agent is made up of two parts. The first part of the drug should contain the loading dose and the second part should be the maintenance dose. The desired response of the drug is achieved by the loading dose (the initial burst dose causes a rapid onset of the pharmacological effect) and the maintenance dose release of the drug is administered at a slow and steady rate (following the zero-order kinetics) to maintain the pharmacological effect of the drug. The rate of maintenance dose at which a certain drug is administered should be equal to the rate of the drug output [2,47]. Therefore, it is necessary to develop an ideal drug delivery system which should have the above-mentioned characteristics. It has been seen that many drug release products cannot be considered as an ideal delivery system [17]. Table 6 enlisted the various marketed CR formulations.

### Evolution of the Controlled Release Dosage Forms

First-generation: The first generation of controlled release dosage form drugs was from 1950–1980. This generation of dosage forms mainly employs four types of mechanisms for drug release, which accelerates the oral and transdermal formulations. The four types of mechanisms are dissolution, osmosis, diffusion, and ion exchange. Diffusion and dissolution-controlled systems are the most commonly used mechanisms of drug delivery. The success of the first generation of drugs is mainly the development of the oral and transdermal routes. With these drugs, the correlation between in-vitro and in-vivo formulation was well understood and there were no biological barriers detected for this generation [63].

Second-generation: These are less successful; unlike the first generation they have formulations for prolonged release using biodegradable polymers for delivering proteins and peptides. During this period, pulmonary delivery systems were developed for delivering insulin. Due to its lesser bioavailability, it is delivered many times higher per dose than is required for the parenteral injection which results in adverse effects. In the last decade of the second generation, nanoparticles that target the gene and the tumour were studied [47].

Third generation: The new technologies for drug delivery are delivery of poorly water-soluble drugs, long-term and non-invasive technology for delivering protein/nucleic acid/peptide, drug delivery to the targeted site using nanoparticles and the drug delivered through self-regulation [47]. Table 7 shows the evolution of controlled drug delivery systems [63,64].

## 8. Concept of Biomaterials in Controlled Drug Delivery

Biomaterials in the drug delivery system help to modulate the pharmacokinetics of the drug. A biomaterial is a substance that has been engineered to interact with biological systems for a medical purpose, either a therapeutic or a diagnostic one. The choice of polymers or biomaterials plays an important role in designing a DDS with defined physicochemical properties and drug release profiles. The different types of biomaterials like polymers, polysaccharides, proteins, lipids and peptides are used in DDS in scales of varying lengths from nano-sized to macro-sized in different routes of applications. Biomaterials should be selected according to the type of formulation, site and route of drug delivery. It should be biocompatible, biodegradable, non-toxic, preferably hydrophilic and mucoadhesive along with optimum mechanical strength. Both non-degradable and biodegradable biomaterials are used in controlled drug delivery. Biodegradable polymers are preferred, as the degradation removes the DDS from the body and helps prevent the accumulation of toxic remnants. In the case of a non-biodegradable biomaterial-based system, if any complication occurs, chelator and reducing agents can be introduced to disintegrate [48].

### Stimuli-Responsive Biomaterials

The biomaterials that can respond to external stimuli that may be physical or chemical are called smart or stimuli-responsive polymers. In the past, polymers have been used to control the release of the cargos that are active that played an utmost import role in the development of nanomedicines. Smart polymers can be divided into two types: Single stimuli-responsive polymer and dual or multiple stimuli-responsive polymers. The single stimulus helps in inducing the protonation and cleavage by hydrolysis (molecular conformational change). This process of induction can be categorized as exogenous and endogenous stimuli. Temperature, electric pulse and magnetic field are the exogenous stimuli. Enzyme concentration, hormone levels, pH and redox potential all are categorized under endogenous stimuli. The polymers of the pH come under the class of photoelectrolysis that have ionizable groups. To control the drug release from the polymers there are two types of strategies. Firstly, the nanocarriers can be used to release the cargos by activating them. Secondly, the polymer of the charged surface can be positive to get internalized by the cells that are targeted [65]. Table 8 lists the various polymers used in the development of controlled release drug delivery systems (CRDDS).

## 9. Nanocarriers in Controlled and Targeted Drug Delivery

Nanocarriers are sub-micron sized particles with a large specific surface area due to which they offer higher loading or dosing per unit volume. They offer improved bioavailability of the drug where and when it is needed (circulate for much longer periods than the drug alone) [70]. They offer efficient navigation in the complex in vivo environment (protects the drug from undue degradation). They achieve the desired therapeutic responsiveness at a much lower dose which reduces adverse effects of the drug. It is easier to tune the surface chemistry of nanocarriers for different drugs and targeting molecules. Sustained and prolonged release of the drug payload along with targeted delivery of the drug can be achieved. Flexibility in forms for diverse routes of drug administration and formulations is possible with nanocarriers [71]. They can be directed not only to specific cell types but even to specific regions of a cell (i.e., the nucleus for gene delivery). Hence enhanced intracellular trafficking of drugs can be achieved with nanocarriers [72].

### 9.1. Need for Targeted Drug Delivery

The drug reaching the tissue that is targeted has to be effective only on the diseased cells without showing any effect on the healthy cells. Nanocarriers have the capacity to increase the concentration of the drug without causing drug toxicity. The supply of the drug to the specified compartments of the tissues within the cells is called cellular and intracellular targets [73]. Nanocarriers are used to deliver the drug to the sites where drug penetration is difficult due to the anatomical barriers. The blood–brain barrier does not allow most of the drug to enter it; it acts as a selective barrier to the brain. By administration of the drugs in nanocarriers, most of the diseases of the central nervous system can be treated as they can cross the blood–brain barrier. The nanoparticle crosses the blood–brain barrier through transcellular or paracellular pathways [74]. The use of nanocarriers in drug delivery systems for targeted tissues has become more popular because the nanocarriers are capable of reaching remote sites and tissues including crossing the blood–brain barrier. Hence, delivering a drug bound with nano-structures or nanocarriers can significantly improve the distribution of drugs in the body to achieve the maximum therapeutic effect.

The targeted drug delivery system is the system of delivering a drug into the body which is characterised by the transportation of a particular drug selectively at a specified diseased site, to bring pharmacological effects to that particular site and minimize adverse effects on the whole body [75]. As discussed, a conjugating drug with a biologically compatible polymer would increase the ease of delivery of the drug by increasing the solubility, minimizing the toxic effects of the drug, and optimizing the duration of the drug effect [76].

### 9.2. Active and Passive Targeting

There can be two modes by which the drug can be targeted, namely, active and passive targeting. In the active mode, the specific marker, which is expressed exclusively in the diseased cells but not in the normal cells, is targeted [75]. This targeting can be accomplished by allowing a molecule to bind with folate receptors that are over-expressed in the diseased cells [73]. For instance, CA-125 is one of the biomarkers that is overexpressed in ovarian cancer and can be targeted for active targeting mechanisms. In passive targeting, the accumulation of the biocompatible polymer at the site of diseased cells depends mainly on the size of the polymers. Due to the presence of leaky junctions of the vessels, the extravasation of the polymers can occur allowing the polymer to reach the diseased site [76]. 

### 9.3. Nanocarriers in Controlled Drug Delivery

#### 9.3.1. Liposomes

These are the colloidal particles formed by combining the amphiphilic phospholipids that enclose an aqueous compartment surrounded by lipid bilayers [77]. The formation of a closed bilayered structure is due to the hydrophobic effect that helps in organizing the amphiphilic molecules that decrease the interactions that are unfavourable between the hydrophobic chains and the surrounding aqueous environment [78]. Depending on the polar head group, phospholipids can exist as phosphatidylcholine and phosphatidylserine. Phosphatidylcholine is commonly used for making liposomes. The size ranges from 25 nm to 200 nm [79]. Sizes larger than 200 nm get cleared by the reticuloendothelial system and thus have short circulation time in the blood. Liposomes are mainly used for tumour cell targeting due to enhanced permeability and retention (EPR) [80], e.g., amphotericin B liposomal injection

#### 9.3.2. Dendrimers

The term dendrimer means a tree and originates from a Greek word because it is similar to the branches of a tree. Dendrimers are symmetrical around a core and have a sphere-shaped three-dimensional structure [81]. They are synthesized from monomers that can be both natural or synthetic [82]. Polyamidoamines (PAMAM) and polypropyleneimines (PPI) are the two types of dendrimers that are used for biomedical applications [83].

#### 9.3.3. Exosomes

Exosomes are nano-sized cell-derived membrane-bound vesicles of 30–100 nm size that are involved in the intercellular transportation of exogenous and endogenous substances. Therapeutic agents, such as small proteins, mRNA or nucleic acid drugs, can be incorporated into exosomes and then delivered to specific types of cells or tissues for targeted drug delivery [84]. They have been very much used and developed rapidly in recent years due to their high ability of internalization with cells. Natural and engineered exosomes are being utilized for the delivery of peptides and genes [85].

#### 9.3.4. Nanoparticles

These are originally solid colloidal particles of less than 100 nm comprising of macromolecules in which drugs can be entrapped or chemically bonded (covalent bond) to attain physical stability of the drug and to achieve controlled release property [86]. Metallic, polymeric, inorganic-clay and solid-lipid nanoparticles are some of the examples. The nanoparticles are used in increasing the therapeutic effect of the drug and can be used in different routes for administration. Most importantly, the nanoparticle can deliver the drug to a difficult-to-reach site. It can execute the controlled release of the drug efficiently and can minimize the adverse effects [72,87,88].

#### 9.3.5. Nanosphere or Nanocapsule

A nanosphere is a matrix type of DDS which is made of an oligomer or/and a polymer [38]. A nanocapsule is a reservoir type of system consisting of an oily core that is surrounded by a shell polymer. Nanocapsules are used for lipophilic drugs and the size can vary from 5 nm to 1000 nm. Nanocapsules offer better protection and stability to the encapsulated drugs [72,89].

#### 9.3.6. Solid-Lipid Nanoparticles

Solid-lipid nanoparticles (SLNs) have emerged as substitutes to conventional colloidal nanocarriers integrating the advantages of polymeric nanoparticles and liposomes while excluding the toxicity. SLNs are spherical nanoparticles of 50–1000 nm in size and made up of lipids that are solid at room temperature, emulsifiers and API [90]. The SLN safety profile is based on biocompatible lipids that are highly tolerable to the lungs and body. SLNs have the potential to incorporate hydrophilic, lipophilic drugs in addition to proteins and nucleic acids which open new frontiers for drug and gene delivery [91]. The phospholipid fatty substances used for SLNs are smaller in size, flexible and biologically compatible, which allows them to pass through minute arterioles and fenestrations without clotting occurring [72].

#### 9.3.7. Nanofibers

Nanofibers are solid fibres of a few nanometers to 1000 nm in diameter that have a higher surface to volume ratio which is well suited for using them as a carrier for drug delivery. The properties of nano-fibres, like diameter, morphology and porosity, can be modulated to obtain a wide range of drug release kinetics [92]. High loading efficiency and spatial distribution of drugs can be achieved with nanofibrous delivery systems [93]. Nanofibers can be synthesized by the electrospinning technique in which patterning could also be done to tune the drug release [94]. Natural nanofibers are extracted from certain bacteria, called bacterial cellulose, and silk fibroin nanofibers are an excellent alternative to synthetic nanofibers in drug delivery [95,96,97,98]. Nanofibrous drug delivery systems are characterized based on their mode of drug release, structure and composition. To obtain immediate drug release, the nanofibrous mesh is made of a suitable polymer with interconnected porous architecture, high specific surface area and high porosity. Drug release from the nanofibers can be modified as prolonged, stimulus responsive and dual-mode/biphasic [99]. Mostly, swellable or biodegradable polymers are used to modify the drug release. Physico-chemical characteristics of the polymer, the process parameters and environmental conditions can significantly affect the drug release kinetics of nanofibers. The nanofibers’ formulation is very complex and is subject to many variables, while at the same time aids to achieve desired drug release kinetics [92].

#### 9.3.8. Polymersomes

Polymersomes are tiny synthetic vesicles that enclose liquid drugs. These are generally made of diblock copolymers as well as polymer–lipid composites that possess enhanced colloidal stability, encapsulation efficiency, membrane characteristics, etc. Polymersomes are more stable than liposomes and have been proved to have lesser toxicity in the body. They can encapsulate both hydrophobic and hydrophilic drugs [72].

#### 9.3.9. Self-Assembled Polymeric Micelles

Self-assembled micelles are composed of amphiphilic polymers that spontaneously self-assemble to form micelles. The hydrophobic segment forms the core and the hydrophilic segment forms the shell. The size of micelles ranges from 10 nm to 100 nm [100]. The core protects the therapeutic drugs from premature degradation. These are useful for tumour cell targeting due to enhanced permeability and retention (EPR). One of the instances is PLK-1 siRNA combined with doxorubicin as a self-assembled micelle used in cancer patients. At a low pH value of 5, these complexes can disassemble and release the drug [101].

#### 9.3.10. Carbon Nanotubes

Carbon nanotubes (CNTs) are cylindrical large molecules consisting of a hexagonal arrangement of graphene sheets (hybridized carbon atoms), which may be formed by rolling and capped with spherical fullerene. CNTs shows unique electrical property due to the delocalized π-electrons in the *z*-axis. CNTs are classified into three types based on the wall number: Single-walled CNTs, double-walled CNTs and multi-walled CNTs. single wall CNTs (SWCNTs) are a cylinder made of a single graphene sheet, while multiwalled CNTs (MWCNTs) are multilayers of rolled graphene sheets [102]. Carbon nanotubes have recently gained importance due to their high surface area which can conjugate with drugs (both molecules and cells), showing higher efficiency and specificity [103]. Until now, carbon nanotubes have been designed for delivering anti-cancer drugs [104]. However, research is being conducted to design carbon nanotubes for other drugs and also to reduce toxicity. From a broader perspective, Carbon nanotubes can be designed to carry proteins, peptides, nucleic acids and drugs to deliver them in different cells and tissues. Functionalized carbon nanotubes are less immunogenic and impart minimal toxicity [105,106].

#### 9.3.11. Nanoemulsions

Nanoemulsions are a heterogeneous system of oil into water (two immiscible liquids) which are stabilized by surfactants or emulsifiers. They are used to carry drugs that are hydrophobic and administered via various routes of administration. They have better stability to flocculation, creaming and sedimentation as compared to conventional emulsions. The larger surface area and other characteristics allows nanoemulsion to deliver a drug efficiently to a specific target site [107].

#### 9.3.12. Hydrogels

Hydrogels are made from water-soluble/insoluble polymers with cross-linked networking. In hydrogels, the drug is dispersed in a glassy polymer which upon contact with water, swells and releases the drug. The release is water penetration and swelling controlled [108]. Hydrogels swell beyond a certain boundary, several folds greater than their actual volume which facilitates polymer chain relaxation and drug diffusion [109]. Hydrogels can offer spatio-temporal control over the release of various therapeutic agents, including macromolecular drugs, small-molecule drugs and cells. Owing to their tunable physicochemical properties, controllable degradability and protecting capability of labile drugs from degradation, hydrogels serve as a carrier to control drug release. The hydrogels when exposed to water open the cross-linked network to open the spaces in between the polymers. The diffusion of the drug depends on the size of the pores and porosity. The drug diffuses freely from a highly porous hydrogel, whereas network erosion is needed for the release of drugs from less porous hydrogels. The temperature, pH and ionic strength are useful in exploiting the swelling of the hydrogel [110,111].

**Figure 32:**
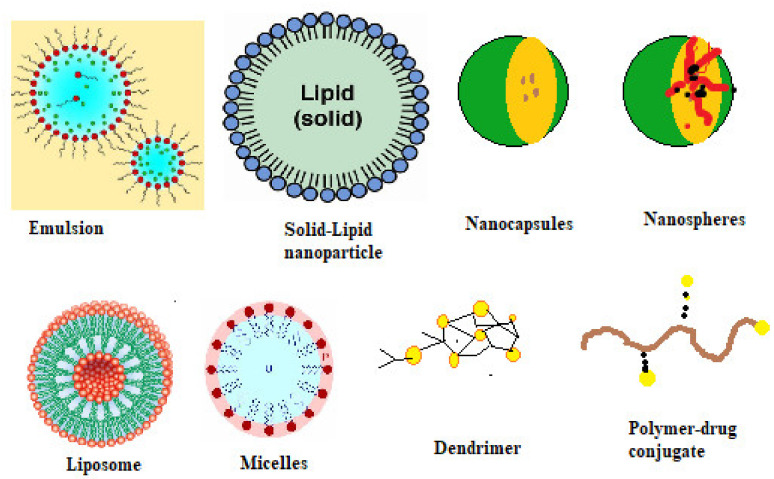
Some common examples of nanocarriers in controlled drug delivery.

Supramolecular hydrogels are three-dimensional cross-linked networks with inter and intra-molecular bonding which offer high water retention capacity, drug loading efficiency and biocompatibility as compared to conventional hydrogels. These hydrogels are mainly useful in self-healing and injectable applications [112]. Bacterial nanocellulose is one such example of supramolecular hydrogel which has been extensively studied in drug delivery in recent times. Interpenetrating network (IPN) hydrogels consist of two or more polymeric networks which are at least partially interlocked on a polymer scale [113,114]. Nanocarriers in controlled drug delivery are schematically shown in Figure 32. The advantages and disadvantages of various nanocarriers in drug delivery are tabulated in Table 9 and Table 10.

## 10. Stimuli-Responsive Drug Delivery Systems Using Smart Biomaterials

Stimuli-responsive drug delivery systems have progressed with the development of biomaterials that are sensitive to external physical environment or stimuli. This is achieved by the incorporation of special functional groups which can influence the chemical, physical and biological properties. These incorporated properties can render the biomaterial responsive to external environmental stimuli [134]. Stimuli-responsive drug delivery systems appear to be a promising approach to controlling and targeting drug delivery. When they are administered, the drug release is activated and then modulated through some action or external input and facilitated by the energy supplied externally. The responsive delivery systems respond to external stimuli such as temperature [135], pH [136], solvent [137], ultrasound [138], electric field [139] and magnetic field [140]. The changes in network structure in response to the external environment are reversible [141].

### 10.1. Chemical Stimuli-Responsive Biomaterials

#### 10.1.1. pH-Responsive

**Figure 33:**
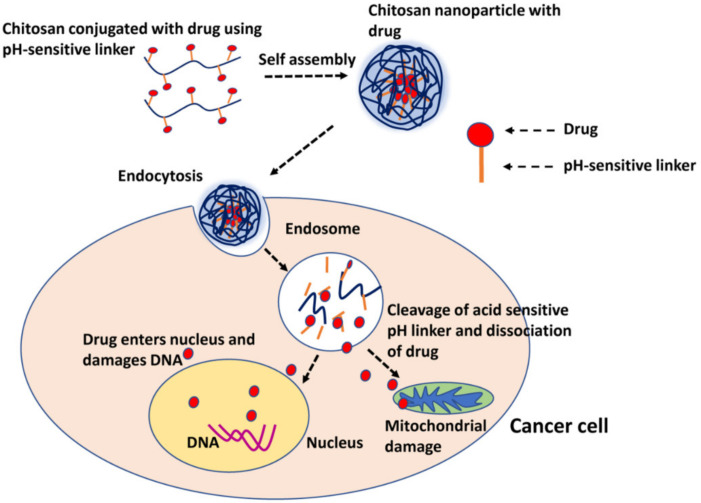
pH-responsive drug release of Tamoxifen from chitosan nanoparticles (adapted from [147] with copyright permission from *Marine Drugs*, MDPI licensed under Creative Commons Attribution (CC BY 4.0) license).

The pH-responsive biomaterials sense the change in pH and undergo physico-chemical changes in polymeric chains which trigger the release of the drug. These are most commonly used for triggering the release of the drug among the other stimuli. The traditionally used pH-responsive carriers show their effects based on the pH of different organs such as the intestine and stomach [142]. pH-responsive polymers can be either polyacids (which sense and release at basic pH) or polybases (which sense acidic pH and release the drug). Examples of pH-responsive polymers are given in Table 7. Eudragit S100 is a citrus-coated pectin nanoparticle that specifically targets the colon of the anticancer drug, 5-Fluorouracil [143]. The carriers that are so designed can differentiate the changes in the pH value at specific sites of the disease like the ischemic tumour sites and inflammatory tissues. They can also be used to differentiate the pH value in different organelles like lysosomes and endosomes. The normal tissues have an extracellular pH of 7.4. In solid tumours, because of an increase in the rate of glycolysis, the pH decreases to 7.0. The low pH of the tumour acts as a stimulus for the controlled drug delivery systems [144,145]. The stimulus of the pH can be combined with other stimuli including redox and temperature to achieve precise release at the specific targets, e.g., poly(2-(diisopropylamino) ethyl methacrylate) (PDPAEMA) [144]. In a recent study, Tamoxifen was loaded onto chitosan-nanoparticles by forming complexes and tamoxifen was released more rapidly at pH 4.0 and 6.0 as compared to pH 7.4, which is a desirable characteristic for tumour-targeted drug delivery [146]. In another study, chitosan nanoparticles were conjugated with an anti-cancer drug using a pH-sensitive linker, which cleaves and releases the drug, after being endocytosed into the cancer cell where the pH is acidic (Figure 33) [147].

#### 10.1.2. Redox Responsive

The change in redox potential triggers the drug release in redox-sensitive biomaterials. These are widely used in the treatment of diseases by use in intracellular drug delivery systems. The redox potential varies in the different tissues in the microenvironments that are useful in designing redox-sensitive drug delivery systems [148]. The designing of the nanoparticles that are glutathione (a redox system in cancer cells) responsive is used in the targeted drug delivery. The glutathione concentration in the normal extracellular matrix is found to be 2–20 μM, while its concentration in the cancer cells is 2–10 mM, which is ten times higher than the normal cells. Due to this difference in the levels of glutathione, it is used as a strategy in designing the controlled drug delivery systems. Some diseased tissue uses the accumulated reactive oxygen which helps in targeting the tissues in the form of reactive oxygen species responsive drug delivery systems. The concentrations of reactive oxygen species are higher in the inflammatory tissues than in the normal tissues; example: ‘‘trimethyl-locked’’ benzoquinone (TMBQ) [149].

#### 10.1.3. Enzyme Responsive

**Figure 34:**
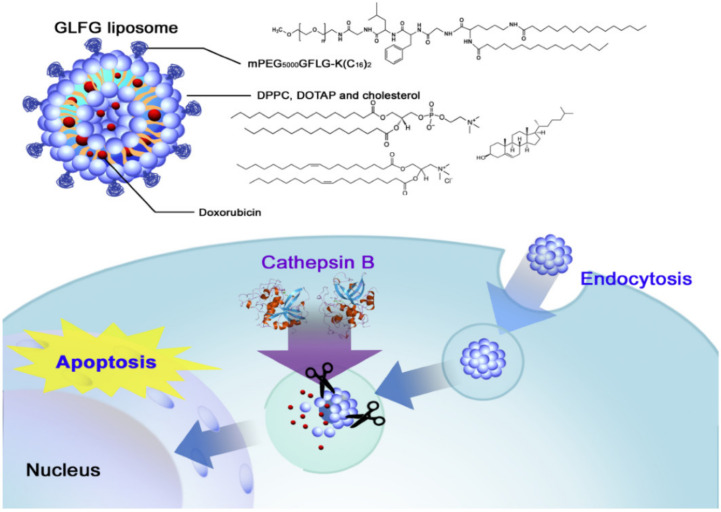
Enzyme-responsive drug release from doxorubicin loaded PEG lipid-GLFG peptide liposome designed as a cathepsin B cleavable peptide linker to hydrolyse and release drugs specifically in tumour cells (reproduced from [151] with permission from *Polymers*, MDPI licensed under Creative Commons Attribution (CC BY 4.0) license).

Here, enzymes are used as triggers in the drug delivery systems. They have unique properties like they are specific to the substrate and are highly selective in cases of mild conditions. As the enzymes are mostly related to the biological and metabolic processes, they can be used in achieving enzyme-mediated drug release at the site of inflammation. The main challenge while using the enzyme-responsive drug delivery systems is that the initial release of the systems has to be controlled precisely. They are named based on their interaction with effector molecules [150]. An example of enzyme triggered release is illustrated in Figure 34. In this study, the anticancer drug doxorubicin was loaded into cathepsin B responsive liposomes made of PEG lipid GLFG-peptide linker. The liposomes are uptaken by endocytosis; in the presence of tumour-specific enzymes (cathepsin B, MMP2/9) and low pH of tumour cell, the lipids get hydrolysed and release the drug [151].

### 10.2. Physical Stimuli-Responsive Biomaterials

#### 10.2.1. Light Responsive

**Figure 35:**
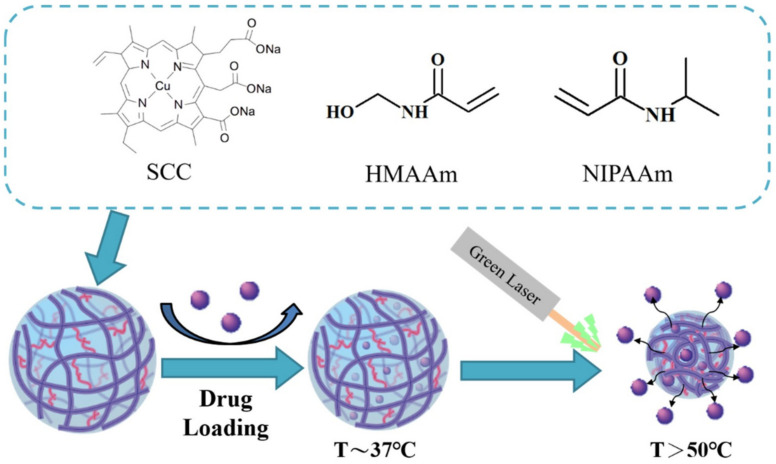
Green laser light induced nanogels (reproduced from [156] with permission from *Polymers*, MDPI licensed under Creative Commons Attribution (CC BY 4.0) license).

This helps in triggering the drug release by the external illumination of light. The photosensitive carriers can release the drug in an on–off system as the nanostructure opens by stimulation of the light. Due to the limitation in the penetration of the light into deep tissues, it restrains the application of the light in a non-invasive manner [152,153]. In a recent study, green laser light was used as a stimulus to heat up and shrink the nanogel for drug release (Figure 35). The elevated temperature and drug release exert an additive effect on cancer cell killing. Liposomes combined with nanoparticles made up of gold can be triggered by light stimulus [154,155].

#### 10.2.2. Thermo-Responsive

**Figure 36:**
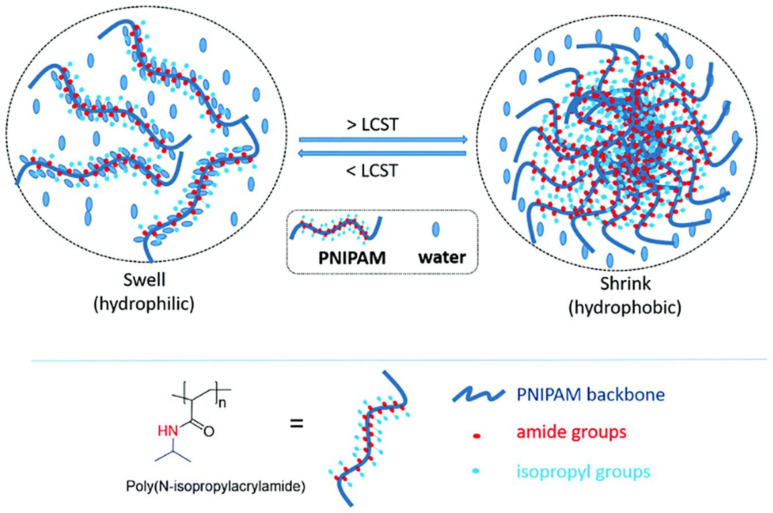
Thermo-responsive drug release by PNIPAM hydrogel (reproduced from [160] with permission from the Royal Society of Chemistry).

Temperature is the stimulus for drug release. Thermo-responsive polymers possess lower critical solution temperature (LCST). Below LCST, polymers are soluble, tend to be hydrated and swell, that is, when drug loading is done. Above LCST, polymers tend to be in a shrunken dehydrated state and the drug gets released. A thermosensitive polymer known as poly(N-isopropyl acrylamide) can exhibit such characteristics [157]. These polymers are found to have hydrophobic groups (methyl or ethyl or propyl). The gelation of 5% polymer solution can become cloudy at 27 °C and on further increase in temperature (at about our body temperature), it forms a gel-like substance. At this physical form (gel-like form), this particular polymer poly(N-isopropyl acrylamide) expels out water from its gel substance [158]. It can revert to its solution state upon a decrease in temperature; see Figure 36. One of the significant advantages of thermosensitive polymers is that they can avoid any organic solvent which is toxic in nature. They also possess the ability to deliver both hydrophilic and lipophilic drugs and at specific sites. They can also deliver the drug at sustained dosage with minimized side effects. Examples could be poly(N-isopropyl acrylamide) as discussed above and poly(methyl vinyl ether) [159].

#### 10.2.3. Electric Responsive

Electric responsive polymers such as polypyrrole [161], Polyaniline [162], poly-imines [163] and graphene [164] are used to fabricate drug delivery carriers. Electro-responsive graphene carriers functionalised with aldehydes (as model drug) through imine-based linkers through covalent bonding and its cleavage upon electrolysis releases the drug [165].

#### 10.2.4. Magnetic Responsive

Magnetic responsive nanoparticles, when applied with high-frequency magnetic field, generate heat. Magnetic nanoparticles are often encapsulated in colloidal carriers including β-cyclodextrins, liposomes, micelles or solid nanoparticles which when exposed to the external magnetic field induce heat and trigger the drug release in cancer hyperthermia. In recent times, core-shell magnetic nanoparticles (i.e., Fe_3_O_4_ and CoFe_2_O_4_) coated with biocompatible polymeric shells (carbohydrate polymers, lignin, polyacids, dextran, etc.) have gained significant importance in cancer therapy [113].

#### 10.2.5. Ultrasound Responsive

**Figure 37:**
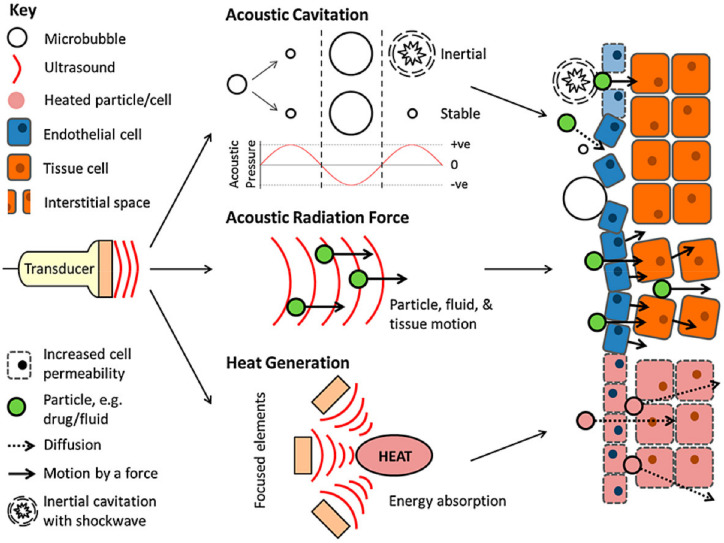
Ultrasound triggered release from microbubbles by mechanical effects by acoustic cavitation and thermal effects by acoustic radiation (reproduced from [167] with permission from *Fluids*, MDPI licensed under Creative Commons Attribution (CC BY 4.0) license).

Ultrasound waves (high frequency > 20 Hz) are used widely for diagnosis as they penetrate deeply into the tissues yet remain safer than X-rays. Ultrasound waves can give 3D images of different organs based on the varied echoes received from different tissues due to the differences in acoustic impedance. Acoustic energy attenuation by the tissues results in fluid streaming, tissue motion and heating which can be used in thermal ablation, transdermal sonophoresis and cavitation [166]. A rapid fall in local pressure causes the vaporisation or evolution of dissolved gases as microbubbles. This helps to disintegrate gall and kidney stones. Ultrasound can be used in combination with pre-existing bubbles or other cavitation nuclei, at lower amplitudes, to harvest a series of mechanical effects that can be exploited for drug delivery [167]. An illustration of this is given in Figure 37.

## 11. Challenges and Future Directions

There has been enormous advancement in controlled drug delivery systems in the past two decades. Nevertheless, there is still scope for advancement to combat the limitations and expand future possibilities.

### 11.1. Nanomedicine Challenges and Improvements

Nano-drug delivery systems have emerged as an excellent alternative to conventional delivery systems with several advantages including targeted drug delivery with enhanced efficacy. However, nanoparticulate systems need to be characterized concerning safety and toxicity. In several studies, nanoparticles resulted in uptake by the reticuloendothelial system and resulted in the inflammation of the liver, lung and brain due to the oxidative stress induced by nanoparticles [168]. The ability of nanocarriers to cross the blood–brain barrier is beneficial in brain diseases; however, it causes neurotoxicity when the intended site of action is not the brain. In addition, nanoparticles provoke immunomodulatory effects in some cases. This immunomodulatory effect of nanoparticles can be harnessed to target inflammatory monocytes across the blood–brain barrier to prevent the progression of auto-immune disorders (e.g., autoimmune encephalomyelitis) [169]. Inorganic mesoporous nanoparticles have gained attention in controlled drug delivery as they comprise ordered mesopores (2–6 nm) and tunable size (50–200 nm) and shape and their easy surface modification makes them ideal for improved targeting and endosomal release of the drugs. To avoid the premature release of drugs through the mesopores, they can be covered with stimuli-responsive polymers, which makes them capable of providing spatio-temporal control during the release of a specific drug into the cytosol of the target cell [170].

On the other hand, stimuli-responsive delivery systems seem to be a very interesting and useful approach to tune the drug release from outside and from within. However, there is a lot more research needed to improve the accuracy, precision and repeatability of such dosage forms. Sensitivity to the specific stimuli must be higher because delivering a high amount of external stimuli (electric field, magnetic field, light and heat) might cause damage to the healthy tissues. Until now, there are no discrete guidelines for nano-drug delivery and stimuli-responsive and functional biomaterials. There is an urgent need to develop and harmonize the regulatory guidelines on nano-drug delivery systems, stimuli-responsive delivery systems and next-generation biomaterials for drug delivery. FDA should establish regulatory guidelines that specifically apply to nanomedicine products, particularly because the safety and toxicity of many nanomaterials have not been fully characterized. Hence, getting regulatory approval for nanomedicine has been very difficult and pharmacoeconomic analysis has to be done before the development.

### 11.2. Microfluidics in Controlled Drug Delivery

Microfluidics systems for implantable and controlled delivery is an interesting field for future research. It is also known as lab-on-a-chip (LOC) technology which involves micro-devices that come with small chambers and channels [171]. These micro-devices control the behaviour of the flow of fluids to deliver the drug to a specific site which is more efficient [67]. Recent studies have suggested the development of synthetic polypeptides by polymerizing α-amino acid N-carboxy anhydrides (NCAs), which can be organized into nanostructures and precisely deliver the drug at a particular site. Moreover, the release of the drug substances can be programmed by manipulating the physical and chemical properties of the polypeptide structure [68]. Antibodies discovery and cell delivery are other significant applications where microfluidics are being employed [172,173].

### 11.3. Molecularly Imprinted Polymers (MIPs)

**Figure 38:**
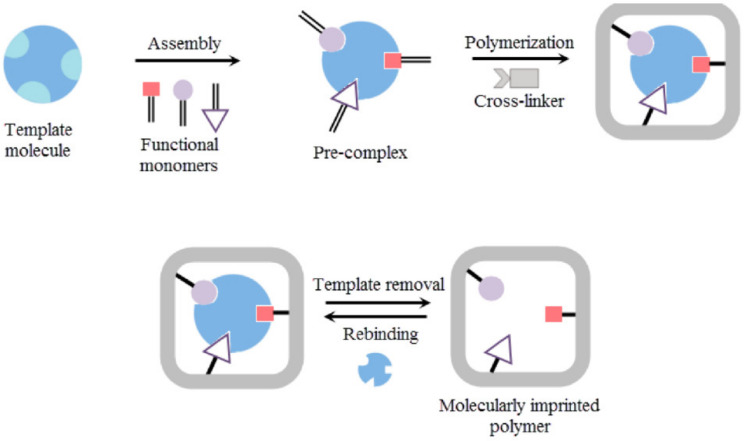
Molecular Imprinting polymers synthesis protocol (reproduced from [176] with permission from *Sensors*, MDPI licensed under Creative Commons Attribution (CC BY 4.0) license).

Molecular imprinting polymers are cross-linked polymers that have binding sites that are specific to the target molecule. These are the cross-linked polymers that have binding sites specific to the target molecule. The molecularly imprinted polymers are developed from five components as the template, cross-linker, porogen, monomer and initiator [174]. The template helps in determining the choice of a functional monomer. It acts as an artificial receptor of target molecules and functions as a biomimetic way of natural antibody-antigen systems. Their mechanism can be understood from lock and key where MIPs selectively bind the molecule with which they were templated during synthesis (Figure 38). MIPs are excellent and promising candidates in developing vaccines and biologic drug delivery as the drug-target specificity can be clearly determined [175].

### 11.4. Intelligent Biomaterials

There is a huge scope for the development of intelligent biomaterials which can sense and auto adapt to the environment and control drug release, for instance, an intelligent hydrogel which can sense the blood sugar levels in the surrounding environment (either pH or temperature) to deliver the specific dose of insulin that is required to maintain the blood sugar levels. There is a need to develop smaller hydrogels but the current challenges that are present in developing smaller biosensor hydrogels are that they are more fragile and sufficient mechanical strength cannot be imparted to fulfil the purpose [68].

### 11.5. CRISPR CAS9 Based Systems

More recently, there has been an increase in attention towards drug release based on CRISPR or clustered regularly interspaced short palindromic repeats are a group of DNA sequences that are mainly found in prokaryotes as an adaptive immune system effector. It has brought revolutionary changes in the science of tissue-specific gene editing [176]. This newly developed delivery system based on CRISPR is composed of sgRNA or single guided RNA and a Cas9 endonuclease. This combination of sgRNA and Cas9 directs the protein (Cas9) to a specific target site based on RNA and DNA. The specific target is recognized by crRNA or CRISPR RNA sequences. However, research is being conducted to minimize the off-target effects brought about by the combination of sgRNA and Cas9 protein. The whole mechanism is quite applicable while delivering any protein drug substance instead of Cas9 [70].

### 11.6. Quantum Sensing Drug Delivery

Another technology that has created a bridge between nanotechnology and drug assay is quantum dots or QDs. These are basically semiconductors of carbon-based nanoparticles of strong chemical inertness, higher specific surface areas, lower capacity to impart toxicity and higher solubility [177]. QDs possess unique optical properties that display quantum confinement effect and emit fluorescence when excited with a light source which makes them a potential candidate for nano-probes and carriers for biomedical application. Most of the drug carriers which are made up of polymers have a limitation of real-time tracing of the drug, which can be achieved by using QDs due to their spectral characteristics. The Fluorescent emission of quantum dots is much better than organic dyes due to which QDs act as a tag for other drug carriers and the drug can easily be traced with the help of quantum dots [177]. Another study reported an RNA delivery approach by combining siRNA and QDs [71].

### 11.7. Three-Dimensional Printing in Drug Delivery

Three-dimensional-printed drug delivery systems have attracted attention in both tissue engineering and drug delivery due to the ability to specifically construct the systems with multiple materials and the unparalleled potential for printing complex physiological structures and organs. The latest innovations in 3D printing offer customized personalized medication for better therapeutic efficacy in customized medical devices, drug-eluting implants and printlets (3D-printed tablets) with a tailored dose, shape, size and release characteristics [178,179].

## 12. Conclusions

The dosage form is a combination of drugs and excipients. Excipients are used to get a structure, enhance stability and mask the taste. Solid, semisolid and liquid dosage forms are the conventional dosage forms that suffer from fluctuations in plasma drug levels which demands high dosing and dosing frequency with poor patient compliance. The bioavailability of a drug is crucial to achieving the desired action from any dosage form. Controlled drug delivery systems have emerged as an alternative to the conventional sort, to improve the bioavailability, extent the drug release and maintain drug plasma levels within the therapeutic window with minimal side effects. Controlled drug delivery increases the drug solubility and stability and offers the selective delivery of drugs with a predictable rate and mechanism to specific organ/tissue/cells. Dissolution, diffusion, water penetration and chemically controlled drug delivery systems are the types of controlled drug delivery systems. Stimuli-responsive delivery systems are useful in various disease conditions (cancer, infections, etc.) to target as well as control the release. Further, nanocarriers with intelligent biomaterials and additive manufacturing techniques can be developed to achieve controlled targeted delivery. The future of drug delivery is focused on patient-specific therapy using microfluidic-based, 3D-printed devices and CRISPR cas9 based delivery systems integrated with quantum sensing.

## Figures and Tables

**Table 1:** Routes of drug administration.

Oral	Swallowed by Mouth as a Tablet, Capsule, Lozenge, or Liquid
Buccal	Held inside the cheek
Sub-lingual	Placed below the tongue
Enteral	Delivered directly into the stomach or intestine
Inhalable	Breathed in through a tube or mask
Nasal	Given into the nose by spray or pump
Ophthalmic	Given into the eye by drops, gel, or ointment
Otic	Given by drops into the ear
Rectal	Inserted into the rectum
Vaginal	Inserted into the vagina
Topical	Applied to the skin
Transdermal	Given through a patch placed on the skin
Infused	Injected into a vein with an IV line and slowly dripped in over time
Intramuscular	Injected into the muscle with a syringe
Intravenous	Injected into a vein or an IV line
Subcutaneous	Injected just under the skin

**Table 2:** Differences between ointment, paste, cream and gel.

Ointment	Cream	Paste	Gel
Hydrocarbon based greasy semisolid	Mostly water-based where drugs are loaded in O/W or W/O emulsion	It is basically an ointment where a high percentage of insoluble solids are added	The liquid phase is trapped within a three-dimensional polymeric matrix
Translucent to opaque	Opaque	Opaque	Transparent
Greasy	Less greasy	Less greasy	Non-greasy

**Table 3:** Advantages and disadvantages of conventional delivery systems.

Advantages of Conventional DDS	Disadvantages of Conventional DDS
Convenience in administration	Poor absorption from site of administration
Non-invasive and better IVIVC	No target specificity
Accurate and measured unit dosage form	Premature excretion from the body
Higher shelf-life	Premature metabolism of the drug
Accommodate patient variation	Poor bioavailability
Flexibility for physician to dose adjustment	Repeated dosing
Low cost	Poor patient compliance

**Table 4:** Advantages and disadvantages of controlled drug delivery systems.

Advantages of Controlled DDS	Disadvantages of Controlled DDS
Controlled or defined drug release	Possible toxicity of materials used
Target specificity	Dose dumping
Long residence of drug	Invasive procedure to implant or remove the system
Protection from metabolism by enzymes/chemicals	Uptake by RES reduces efficacy
Improved bioavailability	Poorer IVIVC
Low dosing frequency	Limited standards
Better patient compliance	Higher manufacturing cost

**Table 5:** Diffusion-controlled reservoir and matrix systems.

Diffusion Controlled Reservoir Systems	Diffusion Controlled Monolithic/Matrix Systems
Easier to achieve zero order	Difficult to achieve zero order
Degradable systems may be difficult to design	Suitable for degradable and non-degradable systems
Rupture can result in dose dumping	No danger of dose dumping
Drug inactivation by contact with the polymeric matrix can be avoided	Not all drugs can be blended with a given polymeric matrix

**Table 6:** Summary of some marketed CR formulations.

Sr. No	Molecule/Drug	Marketed CR Formulation	Manufacturing Company
1	Zolpidem Extended-Release Tablets	Ambien CR	SANOFI AVENTIS
2	Cyanocobalamin Ferrous Fumarate Folic Acid	Fericap CR	Raptakos, Brett & Co. Ltd.
3	Fluvoxamine Extended-Release	Luvox CR	JAZZ PHARMS
4	disopyramide	Norpace CR	Pfizer Laboratories
5	carbidopa 25 mg, levodopa 100 mg	Sinemet CR	Sun Pharmaceuticals
6	Paroxetine Hydrochloride Hemihydrate 12.5 mg	Paxil CR	GSK

**Table 7:** Evolution of Drug Delivery Systems from 1950 to 2040.

Drug Delivery System Size Scale	Macroscale	Microscale and Nanoscale	Nanoscale (Targeted Delivery)
	Implants (e.g., Subcutaneous or intramuscular	Reservoir DDS (e.g., Oral tablets, drug-eluting stents, catheters)	Injected nanocarrier DDS (e.g., PEGylated drugs, PEGylated liposomes, PEGylated polymeric micelles, polymer-drug conjugates
	Inserts (e.g., Vaginal, ophthalmic)	Injected matrix or monolith depots (e.g., Degradable microparticles and phase separation)	
	Ingested DDS (e.g., Osmotic pumps, hydrogels)	Early nanoparticles and PEGylation DDS (e.g., Polymeric micelles and liposomes)	
	Topical DDS (e.g., Skin patches)		
	**1st Generation**	**2nd Generation**	**3rd Generation**
**Drug Delivery System** **Technologies**	**Basics of Controlled Release (1950–1980)**	**Smart Delivery System (1980–2010)**	**Modulated Delivery System (2010–2040)**
	Oral delivery	Zero-order release	On-Off insulin release
	Transdermal delivery	Smart polymers and hydrogels	Targeted delivery
	Drug release mechanism	Peptide and protein delivery	Long term delivery system
		Nanoparticles	In-vitro and in-vivo correlation

**Table 8:** Polymers used in the development of controlled release drug delivery systems.

Synthetic Polymers [66,67]	Natural Polymers [67,68]	Stimuli-Responsive Polymers [69]
Polyhydroxy ethyl methacrylate poly (2-hydroxyethyl methacrylate) Ethyl cellulose Hydroxypropyl methyl cellulose (HPMC) Eudragits Polylactic acid (PLA) Polylactic-co-glycolic acid (PLGA) Polycaprolactone Polyvinyl Pyrrolidone (PVP) Poly methyl methacrylate (PMMA) Poly-(N-Isopropyl acrylamide) (PNIPAM) Poly(ethylenimine) Cyclodextrin (α, β, γ) Carbomers	Alginates Starches Dextrans Cellulose Gums (Acacia, Tragacanth, Guar gum) Chitosan Collagen Gelatine Microbial polymers (Polyhydroxy butyrate) Arginine derivatives	**pH-responsive:** Polyacids (PLA, Polymethacrylate, Poly aspartate, alginates, polystyrene sulphonic acid) Polybases (Chitosan, poly-L-Lysine, Polyallylamine, Poly ethylene amine, Poly amidoamine dendrimer) **Thermoresponsive:** Poly-(N-Isopropyl acrylamide) (PNIPAM) Poly-(N-Vinylcaprolactam) Poly(N,N-dimethyl acrylamide) Poly (methyl vinyl ether) **Electric responsive:** Sulfonated polystyrenes Poly(thiophene)s Poly(ethyl oxazoline)s **Ultrasound responsive:** Ethylene-vinyl acetate **Light responsive:** Modified poly(acrylamide)s

**Table 9:** Advantages and disadvantages of nanocarriers in drug delivery [115,116].

Advantages	Disadvantages
Specificity and targeted delivery of drugs can be achieved	Unintended penetration and translocation of nanocarriers to the blood–brain barrier, lungs results in toxicity
Improved tumour penetration for anticancer drugs	Nanocarriers can change in shape and size resulting in varied physicochemical interactions and activity
Enhanced Permeability and Retention can permit the passive accumulation	Suboptimal delivery due to heterogeneities of nanocarriers in vascular permeability
Enhanced bioavailability and efficacy	Uptake by RES can reduce the efficacy
Controlled delivery of drugs with low dose	Limited availability of animal models

**Table 10:** Advantages and disadvantages of various nanocarriers in drug delivery.

Nanocarrier	Advantages	Disadvantages	Refs.
**Liposomes**	Less cytotoxic Amphiphilic and Self-assembly capability Can load both hydrophilic and lipophilic drugs High payload Longer duration of action	Could crystallize during long term storage Poor control over the drug release rate Lack of means to prevail biological barriers Sufficient loading of drugs for which pH and ion gradients do not apply Leakage and fusion of loaded drug Phospholipids may undergo oxidation and hydrolysis	[117,118]
**Dendrimers**	Uniformity in molecular weight, size, shape and branch length A high degree of branching results in a high surface area Availability of internal cavities with Polyvalency offer high loading and targetting High water solubility Biocompatibility and absence of immunogenicity	Complex synthesis process Possibility of incomplete reactions with terminal groups Steric hindrance to the core molecule and dendrons obstructs the formation of high generation dendrimer	[119,120]
**Exosomes**	Cell targetting anad gene delivery Ability to loading both hydrophilic and lipophilic drugs Exosomes membranes possess many proteins thus show very high organotropism Immunocompatible if derived autologous	Rapid clearance from the blood Current methods available suffer low drug loading and retention Purification and large scale extraction is a hassle	[121,122]
**Metal Nanoparticles**	Tunable sizes and shapes (spherical, triangular, cubic, rods, starts, etc.) Possibilities of easy functionalization Size-dependent activity	RES uptake might result in low biocompatibility and cytotoxicity Instability of nanoparticles	[88,123]
**Mesoporous silica nanoparticles**	Ordered porous structure High surface area Tunable pore size and functionalization Poorly water-soluble drugs and gene delivery	More studies are needed on cytotoxicity The presence of high surface silanol groups interacts with the phospholipids of the red blood cell membranes leads to hemolysis	[124,125]
**Carbon nanotubes**	High surface area, enhanced conductivity and strength Vast functionalization sites Optical properties For targeted delivery	High immunogenicity, carcinogenicity and cytotoxicity Non-biodegradable Poor aqueous solubility and poor absorption	[103,126]
**Nanocapsules/nanospheres**	Efficient drug accumulation at the target site Controlled release of drug over weeks	Non-degradable polymers accumulate in tissues In vivo metabolism and elimination, routes are not elucidated	[127,128]
**Quantum dots**	Semiconductor nanocrystals with broad excitation spectra, narrow emission spectra, tunable emission peaks Possess long fluorescence lifetimes and negligible photobleaching Ability to conjugate with proteins and multiple molecular targets simultaneously	Quantum dot degradation result in the leaching of heavy metals such as Cadmium which generates reactive oxygen species (ROS) High cytotoxicity	[129,130,131]
**Nanofibers**	High specific surface area Multiple drugs with high loading capacity Tunable physicochemical properties Good Spatio-temporal distribution of drugs Great choice of polymers that are biodegradable and biocompatible Designed for various routes of administration for both hydrophilic and hydrophobic drugs	Scalability is an issue Poor control over nanofiber dimensions Need to optimize the solvent system for each polymer in the electrospinning process	[96,132,133,134]

## Abbreviations

**Abbreviation**	**Full form**
API	Active Pharmaceutical Ingredient
FDA	Food and Drug Administration
BCS	Biopharmaceutics Classification System
BBB	Blood Brain Barrier
IVIVC	In vitro In vivo Co-relationship
CYP450	Cytochrome P450
t_1/2_	Biological half-life
TI	Therapeutic Index
TW	Therapeutic Window
MEC	Minimum effective concentration
PK	Pharmacokinetics
ED_50_	Effective dose in 50% of subjects
TD_50_	Toxic dose in 50% of subjects
DDS	Drug delivery systems
CRDDS	Controlled release drug delivery systems
EPR	Enhanced permeability and retention
PLK 1	Serine/threonine-protein kinase
siRNA	Small interfering Ribose Nucleic acid
CNTs	Carbon nanotubes
SWCNTs	Single-walled carbon nanotubes
MWCNTs	Multiwalled carbon nanotubes
MMP	Matrix metallo proteinases
LCST	Lower critical solution temperature
MIP	Molecularly Imprinted Polymers
CRISPR Cas9	Clustered regularly interspaced short palindromic repeats
s*gRNA*	Single guide RNA
LOC	Lab-on-a-chip
QD	Quantun dots
RES	Reticulo Endothelial system
